# Physiological advantages of C_4_ grasses in the field: a comparative experiment demonstrating the importance of drought

**DOI:** 10.1111/gcb.12498

**Published:** 2014-03-28

**Authors:** Samuel H Taylor, Brad S Ripley, Tarryn Martin, Leigh-Ann De-Wet, F Ian Woodward, Colin P Osborne

**Affiliations:** 1 Department of Animal and Plant Sciences, University of SheffieldSheffield S10 2TN, UK; 2 Botany Department, Rhodes UniversityGrahamstown 6139, South Africa

**Keywords:** C_3_ photosynthesis, C_4_ photosynthesis, drought, gas exchange, PACMAD, Poaceae, stomatal conductance, water potential

## Abstract

Global climate change is expected to shift regional rainfall patterns, influencing species distributions where they depend on water availability. Comparative studies have demonstrated that C_4_ grasses inhabit drier habitats than C_3_ relatives, but that both C_3_ and C_4_ photosynthesis are susceptible to drought. However, C_4_ plants may show advantages in hydraulic performance in dry environments. We investigated the effects of seasonal variation in water availability on leaf physiology, using a common garden experiment in the Eastern Cape of South Africa to compare 12 locally occurring grass species from C_4_ and C_3_ sister lineages. Photosynthesis was always higher in the C_4_ than C_3_ grasses across every month, but the difference was not statistically significant during the wettest months. Surprisingly, stomatal conductance was typically lower in the C_3_ than C_4_ grasses, with the peak monthly average for C_3_ species being similar to that of C_4_ leaves. In water-limited, rain-fed plots, the photosynthesis of C_4_ leaves was between 2.0 and 7.4 *μ*mol m^−2^ s^−1^ higher, stomatal conductance almost double, and transpiration 60% higher than for C_3_ plants. Although C_4_ average instantaneous water-use efficiencies were higher (2.4–8.1 mmol mol^−1^) than C_3_ averages (0.7–6.8 mmol mol^−1^), differences were not as great as we expected and were statistically significant only as drought became established. Photosynthesis declined earlier during drought among C_3_ than C_4_ species, coincident with decreases in stomatal conductance and transpiration. Eventual decreases in photosynthesis among C_4_ plants were linked with declining midday leaf water potentials. However, during the same phase of drought, C_3_ species showed significant decreases in hydrodynamic gradients that suggested hydraulic failure. Thus, our results indicate that stomatal and hydraulic behaviour during drought enhances the differences in photosynthesis between C_4_ and C_3_ species. We suggest that these drought responses are important for understanding the advantages of C_4_ photosynthesis under field conditions.

## Introduction

C_4_ photosynthesis is a fascinating example of a complex phenotype that has evolved repeatedly (Sage *et al*., 2011), and influences a suite of ecophysiological traits that determine plant performance in natural settings (Long, 1999). Today, C_4_ grasses are vital as agricultural crops (e.g., maize and sugarcane) and dominate the ground cover over large areas of Africa, Australia, South Asia and the Americas (Edwards *et al*., 2010). The role of climate in determining the relative performance of C_4_ and C_3_ species from both monocot and eudicot lineages is, therefore, a key question in studies of global change (Epstein *et al*., 1997; Murphy & Bowman, 2006; Arnone *et al*., 2011; Morgan *et al*., 2011).

The principal physiological innovation common to C_4_ lineages is the development of a biochemical CO_2_ pump that operates as an extension of the dark reactions of photosynthesis (Hatch & Osmond, 1976). The C_4_ pump elevates CO_2_ concentrations in photosynthesising chloroplasts, virtually eliminating O_2_ competition for the active site of Rubisco and therefore photorespiration, while in C_3_ plants, photorespiration limits net CO_2_ assimilation (*A*) at higher temperatures and low partial pressures of CO_2_ (Osmond *et al*., 1982). The efficient delivery of CO_2_ to Rubisco in C_4_ plants improves photosynthetic efficiency at high temperatures, but bears an energetic cost that limits the maximum efficiency of photosynthesis in C_4_ species at low temperatures (Ehleringer & Pearcy, 1983). The initial CO_2_ assimilation step in C_4_ plants, which is catalyzed by PEP-carboxylase in combination with carbonic anhydrase, also has a higher affinity for its substrate than that of C_3_ plants. This generates the CO_2_ concentrating effect of the C_4_ pump and, in combination with the increased assimilation rates driven by the pump, means that C_4_ leaves are able to maintain higher *A* at lower internal CO_2_ concentrations (Collatz *et al*., 1992). The rates of supply of CO_2_ to the intercellular spaces of leaves and the loss of water through transpiration (*E*) are intrinsically linked, and water use can be limited by reducing stomatal conductance (*g*_s_; Raschke, 1975). C_4_ photosynthesis therefore has important consequences for leaf water-use efficiency, i.e. net CO_2_ assimilation per unit of water loss, a key observation noted in the earliest studies of C_4_ ecophysiology (Black *et al*., 1969; Bjorkman, 1971).

Despite the important consequences of C_4_ physiology for water use, until recently the primary ecophysiological explanation for C_4_ grass species distributions was considered to be growing season temperature (Teeri & Stowe, 1976). For C_4_ eudicots, however, adaptation to arid environments has long been accepted as important in shaping species distributions (Ehleringer & Monson, 1993; Ehleringer *et al*., 1997). For the grass family (Poaceae), within which the majority of C_4_ species and 30% or more of C_4_ evolutionary origins occur (Sage, 2009; Sage *et al*., 2011), habitat water availability and plant hydraulics are receiving renewed attention for their role determining the evolutionary success and distribution of C_4_ species (Edwards & Still, 2008; Osborne & Freckleton, 2009; Edwards & Smith, 2010; Osborne & Sack, 2012; Pau *et al*., 2012; Taylor *et al*., 2012; Griffiths *et al*., 2013). Recent use of molecular phylogenies has provided new insights into how evolutionary processes have shaped species distributions with respect to climate. Occupation of cooler habitats by C_3_ species is now known to be associated with a preference for cooler climates in two (Edwards & Smith, 2010; Visser *et al*., 2014) of the nine monophyletic subfamilies of Poaceae (Grass Phylogeny Working Group II, 2012): Pooideae (Vigeland *et al*., 2013) and Danthonioideae (Humphreys & Linder, 2013), species of which are all C_3_ (Edwards & Smith, 2010). In contrast, comparisons between C_3_ and C_4_ grasses within the PACMAD clade are most appropriate to studies of the adaptive advantages of C_4_ photosynthesis among grasses (Edwards *et al*., 2007; Edwards & Still, 2008). The PACMAD clade includes the monophyletic subfamilies Panicoideae, Arundinoideae, Chloridoideae, Micrairoideae, Aristidoideae and Danthonioideae, excludes the Pooideae, and encompasses the evolutionary origins of all contemporary C_4_ grass species (Christin *et al*., 2009; Grass Phylogeny Working Group II, 2012). Within the PACMAD clade, the evolution of C_4_ photosynthesis has resulted in preferences for drier habitats by C_4_ lineages (Edwards & Still, 2008; Osborne & Freckleton, 2009; Edwards & Smith, 2010; Pau *et al*., 2012), and divergence in water-use traits between C_3_ and C_4_ grasses (Taylor *et al*., 2012; Griffiths *et al*., 2013).

Paradoxically, as evidence has mounted to support the importance of drier habitats to the evolutionary success of C_4_ photosynthesis in grasses, it has become clear that photosynthesis in these species may be more susceptible to failure under declining leaf water status (reviewed by Ghannoum, 2009; Driever & Kromdijk, 2013). Following restriction of watering in pot-based experiments, *g*_S_ of C_3_ grasses declines to a greater degree and C_3_ water-use efficiency can increase to match that of C_4_ plants (Ripley *et al*., 2010; Taylor *et al*., 2011). However, there is evidence that C_3_ grasses commonly operate at more negative leaf water potentials (Ψ) than C_4_ species (Ripley *et al*., 2010; Taylor *et al*., 2010, 2011). As a consequence of these observations, it has been proposed that differences in plant hydraulics may have played an important role in allowing C_4_ grasses to colonize and adapt to dry and open habitats (Osborne & Sack, 2012): decreased responsiveness of Ψ, *g*_s_ and *E* to water availability may result in photosynthesis among C_4_ grasses showing greater resistance to the effects of drought.

To date, observations of susceptibility to drought among C_4_ species have been made primarily in pot-based studies, which have several potential limitations (Poorter *et al*., 2012). There is, therefore, only limited evidence that can be used to compare the impacts of drought on the leaf physiology of closely related C_3_ and C_4_ species under natural growing conditions (Ripley *et al*., 2007; Frole, 2008; Ibrahim *et al*., 2008). Crucially, all of these studies have focused on comparisons within the Panicoideae subfamily, and there is no evidence addressing contrasts across other key PACMAD lineages. We therefore established an outdoor common garden experiment using twelve C_3_ and C_4_ grass species, sampled from four closely related PACMAD lineages. All of the species used in the experiment are found within 60 km of the study site, in a region of the Eastern Cape of South Africa where climate, according to the Koppen–Geiger classification, is warm temperate, fully humid, with warm summers (Peel *et al*., 2007). Our goal was to compare physiological responses of C_3_ and C_4_ grasses to an experimental manipulation of water availability, testing whether responses of leaf gas exchange and water potential previously observed under more controlled conditions are important under natural climatic conditions.

Based on our previous experiments (Ripley *et al*., 2007, 2010; Taylor *et al*., 2010, 2011), we hypothesized that C_4_ grasses would show higher *A*, lower *g*_s,_ and higher water-use efficiency when well watered. During periods of progressive drought, we expected that *g*_s_ in C_3_ grasses would decrease to a greater extent and that differences in leaf water-use efficiency might also diminish between C_3_ and C_4_ grasses (Frole, 2008; Ripley *et al*., 2010; Taylor *et al*., 2011). We further hypothesized that limitation of photosynthesis observed during drought in C_3_ species would be principally driven by decreased *g*_s_, but in C_4_ species would instead be associated with decreased midday leaf water potential (Ψ_midday_; Ghannoum *et al*., 2003; Ripley *et al*., 2007). We also predicted that C_4_ grasses would show less negative Ψ_midday_ and maintain smaller hydrodynamic gradients from soil to leaf (ΔΨ = Ψ_predawn_ – Ψ_midday_) when well watered, differences that we expected to be reduced under drought (Taylor *et al*., 2010, 2011). Finally, we aimed to test whether differences in leaf Ψ were associated with greater plant hydraulic conductance in C_4_ grasses (*K*_plant_ = *E*/−ΔΨ; Osborne & Sack, 2012).

## Materials and methods

### Experimental design and plant species

Twelve grass species of open habitats were drawn from four lineages found in the regional species pool of the Eastern Cape of South Africa (Gibbs Russell *et al*., 1990), based on a random sample of three species per lineage (Table1). The two C_4_ groups were the genus *Aristida* and the tribe Andropogoneae, which share a biochemical subtype (NADP-me) but have independent origins of their C_4_ syndrome (Christin *et al*., 2009; Grass Phylogeny Working Group II, 2012). The C_3_ subfamily Danthonioideae and C_3_ species from the tribe Paniceae were used in comparison; both are important components of grassland ecosystems in southern Africa.

**Table 1:** Details of species used, collection locations and commonly inhabited biome types

Clade (photosynthetic type)	Species	Collection location(S:E; deg,min,sec)	Altitude(m)	Biome description (Gibbs Russell *et al*., 1990)	Number of plants surviving, by treatment, in May 2009	
					Watered	Rain-fed
Panicoideae, Paniceae (C_3_)	*Alloteropsis semialata* ssp. *eckloniana*	33,19,44.54: 26,28,44.21	726	Savanna, Grassland	7	8
	*Panicum aequinerve*	33,19,38.09: 26,31,14.44	660	Grassland, Forest	8	8
	*Panicum ecklonii*	33,19,46.38: 26,28,35.82	714	Grassland	2	4
Panicoideae, Andropogoneae (C_4_)	*Heteropogon contortus*	33,19,11.87: 26,30,29.61	631	Savanna, Grassland, Fynbos, Nama-Karoo	8	7
	*Hyparrhenia hirta*	39,19,06.94: 26,30,37.57	618	Savanna, Grassland, Fynbos, Nama-Karoo	8	8
	*Themeda triandra*	33,17,05.23: 26,29,21.15	639	Savanna, Grassland, Fynbos, Nama-Karoo	7	8
Danthonioideae (C_3_)	*Karoochloa curva*	33,14,54.27: 26,21,26.78	492	Grassland, Fynbos, Nama-Karoo	5	7
	*Merxmuellera disticha*	33,14,54.27: 26,21,26.77	492	Grassland, Fynbos, Nama-Karoo, Afro-Montane	8	8
	*Pentaschistis curvifolia*	33,19,46.38: 26,28,35.83	714	Fynbos	6	6
Aristidoideae (C_4_)	*Aristida congesta* ssp. *barbicollis*	33,13,09.22: 26,37,40.04	487	Savanna, Grassland	7	7
	*Aristida diffusa* ssp. *burkei*	33,14,54.27: 26,21,26.76	492	Savanna, Grassland, Nama-Karoo	8	8
	*Aristida junciformis* ssp. *junciformis*	33,33,44.46: 26,53,39.36	80	Savanna, Grassland, Fynbos	8	8

Plants were collected from field locations (Table1) between January 2007 and January 2008 and established in the outdoor common garden. The common garden had a blocked design, in which individual plots were separated by 2 m of short lawn, and paired 2 × 2 m plots within each of eight blocks were either watered or allowed to receive natural rainfall. Plants were regularly spaced and species locations were randomized within each plot but matched between watered and natural-rainfall plots in each block. All plots were watered on a regular basis until October 2008. After this time, only the plots in the watered treatment received additional water; approximately 28 l (equivalent to approximately 7 mm rainfall) was added to each plot every 2–3 days during the growing season. Following rainfall greater than 10 mm in 48 h, watering was halted for 2 weeks. Plots were weeded and the surrounding lawn mown on a regular basis.

### Weather

Air temperature, humidity, wind-speed and direction, precipitation and photosynthetic photon flux density (PPFD) were recorded using a weather station. This comprised a datalogger (DL2e Delta T, Cambridge, UK); two relative humidity and temperature sensors (RHT2 nl, Delta T) positioned at 0.5 and 2 m; an anemometer (AN4, Delta T) positioned at 2 m; a rain gauge (RG2, Delta T) and a quantum sensor (QS2, Delta T).

### Estimation of reference crop evapotranspiration

To assess the effects of our watering treatment, R Language and Environment version 3.0.1 (R Core Team, 2013) was used to calculate reference crop evapotranspiration (ET_0_, mm day^−1^), which was compared with rainfall and watering inputs. Daily mean values (mean of maximum and minimum) from weather station measurements were used in combination with the Penman–Monteith equation, following Allen *et al*., (1998; Data S1). The method assumes an extensive surface of growing, green grass, completely shading the ground and not short of water. Water shortage was observed at our site and bare soil was maintained between plants, thus the calculated ET_0_ is an approximate guide of true evapotranspiration.

### Leaf water potential

To assess plant water deficits, Ψ_midday_ and Ψ_predawn_ were measured and ΔΨ was estimated as the difference between them, assuming Ψ_predawn_ was equilibrated with Ψ_soil_. Measurements of Ψ_midday_ were paired with measurements of gas exchange (below). For measurement, leaves were enclosed in polythene and immediately excised using a razor blade. The balancing pressure was determined using a Scholander-type pressure bomb. Ψ_predawn_ of leaves selected using similar criteria to those used for Ψ_midday_ was determined before sunrise within 48 h. If rainfall occurred between the collection of midday and predawn measurements, Ψ_predawn_ measurements were either discarded or repeated the following day to better represent prevailing daytime conditions.

### Leaf gas exchange

Gas exchange measurements were made during the final 2 weeks of each month during the growing season. Measurements were made under all but wet and extremely overcast conditions to obtain representative snapshots of seasonal gas exchange. During each day on which leaf gas exchange was measured, measurements were taken for one block between 09:30 h and 15:00 h. The first treatment to be measured was rotated each day, and the order of sampling between species was determined by their randomized positions within each plot.

A portable open gas exchange system (LI-6400; LI-COR, Inc., Lincoln, NE, USA) was used for gas exchange measurements, equipped with a CO_2_ mixer (LI-6400-01) and 30 mm × 20 mm chamber/red-blue LED light source (LI-6400-02B). The CO_2_ mole fraction of air entering the chamber was maintained at 400 *μ*mol mol^−1^. Light levels were matched to a PPFD sensor (LI-190); attached via a 1.5 m extension lead and mounted prior to measurements in each plot, in an unshaded, north-facing position, at 45 ° from vertical and roughly 30 cm above the soil surface. Air temperature in the chamber was not controlled, but the equipment was shaded to prevent excessive heating and to allow the chamber temperature to track that of the air. Leaf temperature was estimated using an energy balance calculation. Incoming air was not scrubbed of water vapour.

As the leaves of most species were narrow (1 to 3 mm wide), multiple leaves were usually inserted into the chamber, with a minimum of 100 mm^2^ total projected leaf area used for all measurements. Leaves selected for gas exchange were the youngest fully emerged leaves on their tillers, with flowering tillers being avoided wherever possible and sections of canopy where leaf blades were exposed to full sun being preferred. Leaf area was calculated based on the known dimensions of the chamber and the combined widths of the inserted leaves at either edge of the chamber, measured using a ruler. Low fluxes were encountered regularly, especially during dry periods, forcing the use of flow rates down to 100 *μ*mol s^−1^ to obtain resolvable differences in CO_2_ (ΔCO_2_  > 10 *μ*mol mol^−1^) and H_2_O (ΔH_2_O > 1 mmol mol^−1^) between the reference air-stream and the chamber. The chamber was tested for leaks by exhaling around the seals immediately after inserting leaves. Measurements were taken as soon as the predicted intercellular CO_2_ concentration (*c*_i_) stabilized. If *c*_i_ failed to stabilize within 3 min, if ΔCO_2_ < 10 *μ*mol mol^−1^, or if leaves being measured were thick/rolled, the chamber was re-tested for leaks and, if necessary, the seal on the chamber was re-adjusted before re-commencing measurements. In all cases where ΔCO_2_ was < 10 *μ*mol mol^−1^, reference and chamber gas analyzers were matched prior to measurement.

For the first set of measurements in November 2008, rolled leaves were routinely unrolled to take measurements. Paired measurements, taken with leaves first rolled and then unrolled, indicated that by unrolling leaves, values for *c*_i_ were elevated to an unusual degree due to increases in estimated *g*_s_ (data not shown). Thus, from December 2008 onwards, tightly rolled leaves were not unrolled during gas exchange measurements.

### Estimation of leaf transpiration

To assess water use at the leaf level, a model implemented in R Language and Environment version 3.0.1 (R Core Team, 2013) was used to estimate *E* for individual leaves from each species in the study. The Penman–Monteith equation (Penman, 1948; Monteith, 1965) was combined with an iterative approach to modelling of leaf energy balance for a horizontal leaf suspended over a lawn (Jones, 1992; Data S2). The model was parameterized using leaf widths based on published values for each species (Data S2), mean values for climate variables (Data S3) and *g*_s_ (Data S4) from each measurement period during the growing season.

### Statistical analysis

Statistical analyses were carried out using the R Language and Environment, version 3.0.1 (R Core Team, 2013). To determine the effects of the watering treatments, a Wilcoxon signed rank test was used to test for differences in weekly ET_0_ − (watering+rainfall) values.

Linear mixed effect models of seasonal changes in physiological traits were fitted using maximum likelihood, and tested for significance using tools in the *lme4* package (Bates *et al*., 2013). The data used in models were species mean values calculated for each month  × treatment combination. Prior to analysis, mean values based on ≤ 2 replicates were eliminated from the dataset and, to improve balance in the dataset, species means that were unpaired across treatments in any given month were also removed. The full datasets used for analysis are plotted in Data S5. Average values for C_3_ and C_4_ groups in both treatments during each monthly sampling interval were predicted as fixed effects. Clade was modelled as a random effect dependent on the month of sampling. Model validation was carried out by inspection of residuals and, except for the model of instantaneous water-use efficiency *(A/E)*, log-transformation was used to improve homoscedasticity of data. Bootstrapped 95% confidence intervals for fixed effect predictions were generated using 1000 simulations of each model.

## Results

### Weather and effect of watering

**Figure 1:**
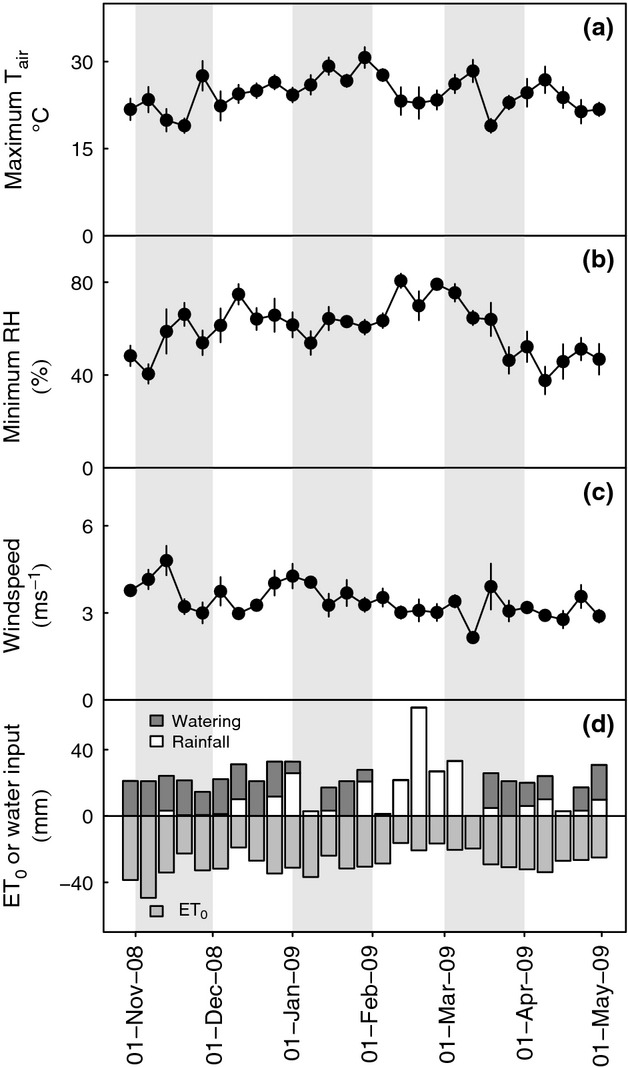
Climate conditions relevant for midday photosynthesis in the common garden experiment carried out in Grahamstown, Eastern Cape of South Africa, during November 2008–April 2009. Weekly values (mean ± SEM) for: (a) daily maximum temperature; (b) daily minimum relative humidity; (c) daily mean windspeed. Weekly totals (d) for water added to the supplementary water treatment, rainfall, and reference crop evapotranspiration calculated using micrometerological data (ET_0_, shown as negative values). Months in the experiment are highlighted by grey-filled areas.

Maximum temperatures were observed in January (mean of daily maxima during January, 28 °C), whereas rainfall and relative humidity were greatest in February (total rainfall, 139 mm; relative humidity, mean of daily minima February 74%; Fig.1a). Relative humidity was lowest during early November (mean of daily minima November 1st–15th 53%) and late March–early April (mean of daily minima March 15th–April 15th 47%; Fig.1b).

Supplementary watering significantly reduced the cumulative water deficit, indicated by rainfall deficit, ET_0_−(watering + rainfall), on a week-by-week basis (Wilcoxon signed rank test, *P* < 0.001; windspeeds used to calculate ET_0_ are shown in Fig.1c). The total accumulated water deficit in the rain-fed plots was estimated to have been more than 3 times that in the plots receiving supplementary water (differences in water input are shown in Fig.1d). Rainfall peaked during the week ending February 19th (Fig.1d). In the 16 weeks prior to the peak of rainfall, total deficits in the watered treatments were 155 mm, compared with 386 mm in the rain-fed treatments. Peak rainfall in February was followed by a further period in which rainfall was low: rainfall deficits were 120 mm in the rain-fed plots and 15 mm in the watered plots over the final 10 weeks of the experiment. We note, however, that as an approximation of soil water balance, rainfall deficit calculated in this manner does not account for soil hydrology and depends on the method used to estimate ET_0_.

### Plant survival

A number of plants died during the 2008–2009 growing season (Table1). The small sample size meant that there was no clear evidence that mortality for any species differed between the watered and rain-fed plots (Table1). Compared with nine deaths in the rain-fed plots, 14 plants died in the watered plots, but six of the dead plants in watered plots were of a single species, *P. ecklonii*. This was one of three species for which more than two of the 16 planted individuals died; *P. ecklonii* (ten dead), *K. curva* (four dead), and *P. curvifolia* (four dead), are all C_3_ plants. Overall, therefore, 19 C_3_ plants died, compared with four C_4_ plants.

### Leaf water potentials

**Figure 2:**
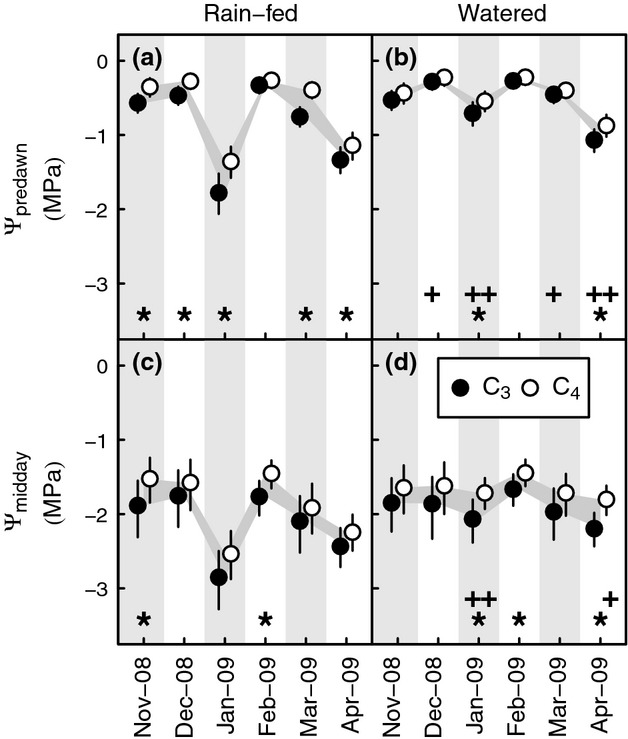
Seasonal contrasts in leaf water potential between C_3_ and C_4_ PACMAD grasses in a common garden experiment at Grahamstown, Eastern Cape of South Africa, between November 2008–April 2009. (a,b) Predawn water potential, Ψ_predawn_; (c,d) midday water potential, Ψ_midday_. Points represent pooled means and 95% confidence intervals for 4–6 species in plots that were rain-fed (a,c) or given supplemental water (b,d). Values are back-transformed from the log-transformed scale used for statistical analysis. Differences between photosynthetic types and months in the experiment are highlighted by grey-filled areas. Photosynthetic type comparisons for which confidence intervals indicate significance at the *P* < 0.05 level are highlighted by * within each pane. Significant differences within photosynthetic types that resulted from watering are indicated by + below the relevant means in (b) and (d).

Leaf water potentials in rain-fed plots were significantly more negative than in watered plots. Watering caused significant increases in average Ψ_predawn_, of 0.26–1.07 MPa, for both C_3_ and C_4_ photosynthetic types during January and April (Fig.2a,b). In December and March, watering led to significant improvements in average Ψ_predawn_ for C_3_ grasses (increase by 0.18–0.29 MPa), but smaller improvements for C_4_ grasses (0–0.05 MPa) were not statistically significant (Fig.2a,b).

Mean Ψ values were always more negative for the C_3_ than C_4_ groups (Fig.2). Except during the wettest month, February, differences in Ψ_predawn_ between the photosynthetic types in rain-fed plots (0.19–0.42 MPa) were statistically significant (Fig.2a). These differences were eliminated in the watered plots, except when drought was most acute during January (0.16 MPa difference) and April (0.19 MPa difference, Fig.2b).

Significant differences in Ψ_midday_ (Fig.2c,d) did not entirely mirror the pattern of drought response shown by Ψ_predawn_. Significant positive effects of watering on Ψ_midday_ were observed for C_4_ grasses in January (0.82 MPa) and April (0.43 MPa; Fig.2d). However, watering significantly increased Ψ_midday_ among C_3_ plants only in January (0.79 MPa), not in April (0.24 MPa; Fig.2d); a contrast with Ψ_predawn_ which was affected consistently across the photosynthetic types at the two timepoints. During the wetter month of February, differences between the average Ψ_midday_ of C_3_ and C_4_ grass leaves were 0.22–0.31 MPa and were significant in both rain-fed and watered plots (Fig.2c,d): these differences were particularly notable, given the lack of differences in Ψ_predawn_ in February (Fig.2a,b). In addition, Ψ_midday_ differed significantly between the photosynthetic types in the rain-fed plots during November. In the watered plots during January and April (Fig.2c,d), significant differences in Ψ_midday_ between the photosynthetic types were coincident with significant effects of watering on Ψ_midday_ of one or both types (Fig.2c,d).

### Gas exchange

**Figure 3:**
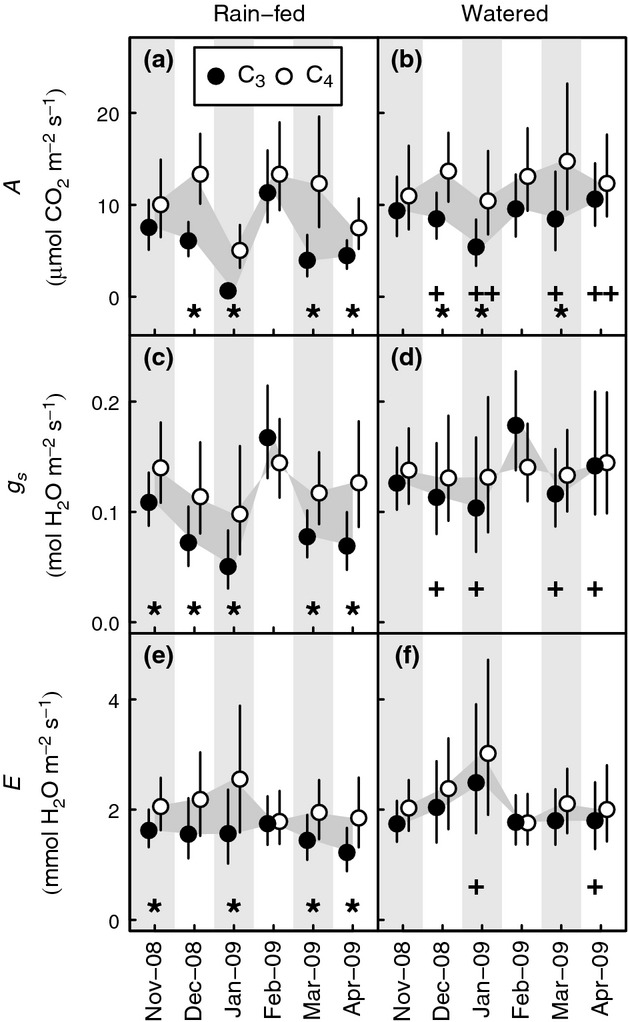
Seasonal contrasts in leaf gas exchange between C_3_ and C_4_ PACMAD grasses in a common garden experiment at Grahamstown, Eastern Cape of South Africa, between November 2008 and April 2009. (a,b) Net CO_2_ assimilation, *A*; (c,d) stomatal conductance to H_2_O, *g*_s_; (e,f) transpiration, *E*. Experimental plots were rain-fed (a,c,e) or given supplemental water (b,d,f). Points represent pooled means and lines 95% confidence intervals for 4–6 species, values are back-transformed from the log-transformed scale used for statistical analysis. Differences between photosynthetic types and months in the experiment are highlighted by the grey-filled areas. Photosynthetic type comparisons for which confidence intervals indicate significance at the *P* < 0.05 level are highlighted by * within each pane. Significant differences within photosynthetic types that resulted from watering are indicated by + below the relevant means in (b), (d) and (f).

In each month, average *A* was always higher among C_4_ grasses (range of means 5.1–14.7 *μ*mol m^−2^ s^−1^) than among C_3_ grasses (range of means 0.6–11.5 *μ*mol m^−2^ s^−1^). In the rain-fed treatment, differences between the photosynthetic types during the drought periods, December–January and March–April, ranged between 3.1 and 8.2 *μ*mol m^−2^ s^−1^, and confidence limits indicated that they were statistically significant (Fig.3a). In the wettest month, February, differences between the photosynthetic types were smaller in the rain-fed plots and were not significant (1.7 *μ*mol m^−2^ s^−1^). This was also true at the start of the growing season in November (2.4 *μ*mol m^−2^ s^−1^; Fig.3a). During this first month of the experiment water deficits in the rain-fed plots may still have been establishing, as watering ceased during October.

In the watered plots, there were also significant differences in *A* between the two photosynthetic types during December, January and March (Fig.3b), which ranged from 4.9 to 6.2 *μ*mol m^−2^ s^−1^. Both photosynthetic types showed significant increases in *A* (4.8–6.0 *μ*mol m^−2^ s^−1^) in response to watering during the most severe drought periods (January and April, Fig.3b), but only the C_3_ group increased *A* significantly in response to watering under less severe drought during December (increase of 2.3 *μ*mol m^−2^ s^−1^) and March (increase of 4.5 *μ*mol m^−2^ s^−1^; Fig.3b). Watering had no significant effect on *A* in the wettest month, February, or in November, at the start of the growing season (Fig.3b).

Unexpectedly, in the natural-rainfall treatment, average *g*_s_ among C_3_ species was often significantly lower, by 0.031–0.056 mol m^−2^  s^−1^, than among C_4_ species (Fig.3c); the exception was the wettest month, February, during which average *g*_s_ among C_3_ species was slightly, but not significantly greater than among C_4_ species (0.022 mol m^−2^ s^−1^, Fig.3c). Differences in average *g*_s_ between the photosynthetic types in the watered treatment were never significant, but showed a similar pattern to those in the rain-fed plots (Fig.3d); the average *g*_s_ of C_4_ species was greater by 0.001–0.028 mol m^−2^ s^−1^, except during February when the C_3_ value was 0.038 mol m^−2^ s^−1^ greater than for C_4_ species. Similar values for *g*_s_ between the photosynthetic types in the watered plots were a result of significant increases in mean *g*_s_ among C_3_ species in December, January, March and April, relative to rain-fed plots, of 0.039–0.075 mol m^−2^ s^−1^ (Fig.3d); watering did not significantly influence the mean *g*_s_ among C_4_ species (Fig.3d). These results contrasted with our expectation that well-watered C_3_ plants would show significantly higher *g*_s_ than their C_4_ relatives.

As expected, patterns in modelled *E* were broadly consistent with the patterns seen for *g*_s_ (Fig.3e,f). Average values of *E* were 0.04–0.64 mmol m^−2^ s^−1^ higher for C_4_ species when compared with C_3_ species in rain-fed plots, and significantly so in November, January, March and April (Fig.3e). Watering eliminated these differences in February, and the maximum difference between photosynthetic types in the watered plots was 0.54 mmol m^−2^ s^−1^ (Fig.3f). The smaller difference in *E* between C_3_ and C_4_ species in the watered plots resulted from watering-induced increases of 2–59% in *E* among C_3_ species. Differences in average *E* in the rain-fed plots were also eliminated during the wettest month, February (Fig.3e). However, in contrast with patterns in *g*_s_, where watering had a significant influence on values in four of six months, watering had a significant effect on *E* among C_3_ species only in the driest months, January and April (Fig.3d,f).

To summarize, although watering always eliminated the differences in *g*_s_ and *E* between photosynthetic types under rain-fed conditions (Fig.3d,f), the differences in *A* between the photosynthetic types persisted in both watered and rain-fed treatments during drought (Fig.3b). Thus, C_4_ grasses held a photosynthetic advantage over their C_3_ relatives when operating at similar *E* and *g*_s_. Furthermore, while decreased photosynthesis among C_3_ species was associated with significant declines in *g*_s_ and Ψ_predawn_ due to drought, the same was not true for their C_4_ counterparts. Among C_4_ species, *g*_s_ and *E* were not significantly affected by water supply, and decreases in *A* coincided instead with significant decreases in Ψ_predawn_.

### Water-use efficiency

**Figure 4:**
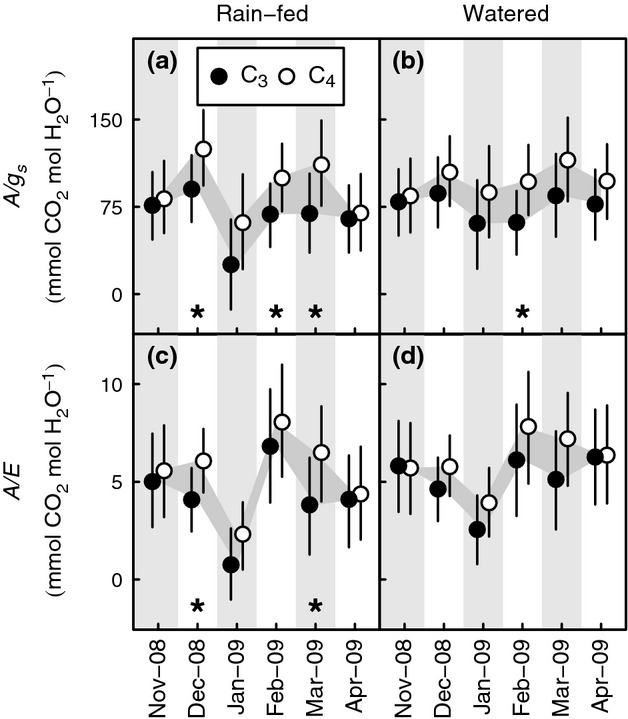
Seasonal contrasts in leaf-level water-use efficiency between C_3_ and C_4_ PACMAD grasses in a common garden experiment at Grahamstown, Eastern Cape of South Africa, between November 2008 and April 2009. (a,b) Intrinsic water-use efficiency, *A/g*_s_; (c and d) instantaneous water-use efficiency, *A/E*. Points represent pooled means and 95% confidence intervals for 4–6 species in plots that were rain-fed (a,c) or given supplemental water (b,d). Values are back-transformed from the log-transformed scale used for statistical analysis (a,b), analysis was carried out on untransformed data (c,d). Differences between photosynthetic types and months in the experiment are highlighted by the grey-filled areas. Photosynthetic type comparisons for which confidence intervals indicate significance at the *P* < 0.05 level are highlighted by * within each pane. There were no significant differences due to watering.

Although leaf-level water-use efficiency tended to be higher, on average, among C_4_ grasses, differences between the photosynthetic types during each monthly sampling interval were rarely significant (Fig.4). At the beginning (November) and end (April) of the growing season, differences in intrinsic water-use efficiency (*A*/*g*_s_) between the photosynthetic types in rain-fed plots were small (6–7 mmol mol^−1^) and were not significant (Fig.4a). Throughout the remainder of the growing season, average *A*/*g*_s_ of C_4_ leaves in rain-fed plots was 64–125 mmol mol^−1^, greater than the values for C_3_ leaves (26–91 mmol mol^−1^) and significantly so during periods of intermediate drought stress in December and March (Fig.4a). This *A*/*g*_s_ advantage to C_4_ grasses in rain-fed plots was therefore at its maximum when *A*, *g*_s_ and Ψ_predawn_ were water limited (i.e. significantly affected by the watering treatment) among C_3_ but not C_4_ grasses (Fig.3). When drought was most severe during January, however, although the difference in *A*/*g*_s_ between the photosynthetic types in the rain-fed plots was large (38 mmol mol^−1^) it was not significant. In contrast, during the wettest month, February, differences of a similar size to that seen in January were significant in both watered (33 mmol mol^−1^) and rain-fed (37 mmol mol^−1^) plots (Fig.4a). To summarize, advantages to the C_4_ grasses in *A*/*g*_s_ were largest in well-watered soil and during mild drought, but were lost under severe drought and were also small at the beginning and end of the growing season (Fig.4a,b).

Variation over the growing season and across treatments meant that average instantaneous water-use efficiency (*A*/*E*) ranged from 0.72 to 6.89 mmol mol^−1^ for C_3_ and 2.29 to 7.99 mmol mol^−1^ for C_4_ plants, and was 1.10–2.61 mmol mol^−1^ greater among C_4_ grasses from December to March. Differences in *A*/*E* were similar to *A*/*g*_s_ in that they usually favoured C_4_ grasses and that in the rain-fed treatment they were smallest in November and April (0.14–0.5 mmol mol^−1^). Indeed, C_3_ grasses showed very similar *A*/*E* to their C_4_ relatives in the watered plots (−0.1–0.06 mmol mol^−1^ difference; Fig.4c,d) in November and April. However, statistically significant advantages to C_4_ grasses were observed for *A*/*E* in the rain-fed plots in December and March (Fig.4c), consistent with significant differences in *A*/*g*_s_. We were surprised to find that, in contrast with *A*/*g*_s_, having accounted for the effects of leaf energy budget by calculating *A*/*E*, differences in leaf instantaneous water-use efficiency between the photosynthetic types were not significant in either treatment during February (Fig.4c,d), the wettest month in the study.

### Plant hydraulics

**Figure 5:**
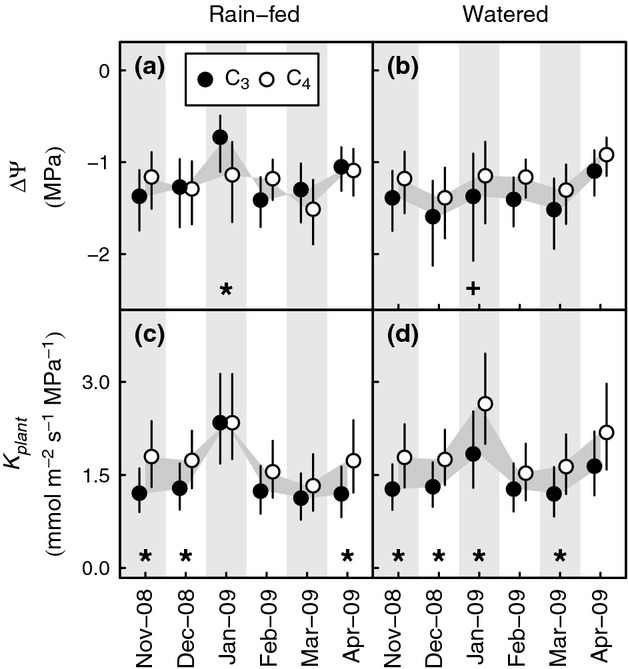
Seasonal contrasts in plant hydraulics between C_3_ and C_4_ PACMAD grasses in a common garden experiment at Grahamstown, Eastern Cape of South Africa, between November 2008 and April 2009. (a,b) Hydrodynamic gradient, ΔΨ* = *Ψ_midday_ − Ψ_predawn_; (c,d) hydraulic conductance, *K*_plant_ = *E/*−ΔΨ. Points represent pooled means and 95% confidence intervals for 4–6 species exposed to natural rainfall (a,c) or given supplemental water (b,d). Values are back-transformed from the log-transformed scale used for statistical analysis. Differences between photosynthetic types and months in the experiment are highlighted by the grey-filled areas. Photosynthetic type comparisons for which confidence intervals indicate significance at the *P* < 0.05 level are highlighted by * within each pane. The significant difference within the C_3_ photosynthetic type that resulted from watering is indicated by + below the relevant mean in (b); there were no significant differences due to watering in (d).

The size of ΔΨ reflects the hydraulic balance between water loss from the leaves and supply from the roots and soil, with more negative values for individual plants indicating greater strain. Average values by photosynthetic type ranged between −0.73 and −1.59 MPa. In watered plots, although differences in ΔΨ between C_3_ and C_4_ species were never significant (Fig.5b), average values for C_4_ species were consistently smaller (−0.91 to −1.38 MPa) than those for C_3_ species (−1.10 to −1.59 MPa; Fig.5a,b). In rain-fed plots, average values for C_3_ species were similar to or smaller than those for C_4_ plants during drought in December (C_3_ −1.27 MPa, C_4_ −1.29 MPa), March (C_3_ −1.30 MPa, C_4_ −1.51 MPa) and April (C_3_ −1.04 MPa, C_4_ −1.08 MPa), differences that were not statistically significant (Fig.5a). The only significant differences in ΔΨ were observed under the most severe drought in January, when the C_3_ ΔΨ (−0.73 MPa) was significantly smaller than both C_3_ grasses in the watered plots (−1.39 MPa) and C_4_ grasses in the rain-fed plots (−1.14 MPa; Fig.5a). This significantly smaller value of average ΔΨ among C_3_ grasses was observed during a period of acute leaf water deficit (Fig.2). Therefore, the nonsignificant changes in a similar direction, which were observed in December, March and April, might also be interpreted as indicative of reduced or more variable hydraulic performance among C_3_ species.

A measure of whole-plant leaf-specific hydraulic conductance (*K*_plant_, mmol m^−2^ s^−1^ MPa^−1^) is provided by the flux of water due to *E* (mmol m^−2^ s^−1^) normalized by ΔΨ (MPa). *K*_plant_ was almost always greater among C_4_ species (1.32–2.69 mmol m^−2^ s^−1^ MPa^−1^) than C_3_ species (1.12–2.35 mmol m^−2^ s^−1^ MPa^−1^; Fig.5c,d), a difference of 0.2–0.88 mmol m^−2^ s^−1^ MPa^−1^. The exception to the general rule that *K*_plant_ was greater among C_4_ species was in the rain-fed plots during January (Fig.5c), when the difference was almost zero (Fig.5a), coincident with the significant decline in average ΔΨ for C_3_ species. Differences in *K*_plant_ between C_3_ and C_4_ species were significant in November, December and April in the rain-fed plots (Fig.5c) and in November, December, January and March in the watered plots (Fig.5d), but watering had no significant effects on *K*_plant_ (Fig.5d).

## Discussion

Our results demonstrate that plant water relations play a key role in maintaining the physiological advantages of C_4_ over C_3_ PACMAD grasses under field conditions. We found that *A*, *E* and *g*_s_ among C_3_ grasses declined significantly in response to drought, in concert with reductions in ΔΨ. In contrast, C_4_ grasses maintained ΔΨ, *E* and *g*_s_ throughout the growing season, and *A* was limited by water supply only under the most extreme drought conditions when both Ψ_predawn_ and Ψ_midday_ decreased. The findings that *g*_s_ is more sensitive to drought among C_3_ grasses and that *A* is more obviously associated with Ψ_midday_ than with *g*_s_ in C_4_ grasses are consistent with evidence from previous experiments (Ghannoum *et al*., 2003; Ripley *et al*., 2010; Taylor *et al*., 2011). That C_4_ gas exchange was relatively independent of water supply and that *g*_s_ among C_3_ species was commonly lower than among C_4_ relatives are novel findings that highlight the importance of both taking a field-based approach and monitoring performance throughout a growing season. While our measurements of ΔΨ and estimates of *K*_plant_ provide some support for the hypothesis that *K*_plant_ is often higher among C_4_ grasses, these differences were not clear-cut, and their physiological basis remains unclear. Therefore, important questions remain about the causes of C_4_ resistance to drought.

We found that *g*_*s*_ among C_4_ plants was independent of our watering treatment, but *A* decreased in conjunction with Ψ_midday_ under more severe drought. Among C_3_ species, decreases in photosynthesis were paired with decreases in Ψ_predawn_ and *g*_s_, but among C_4_ species, decreases in Ψ_predawn_ occurred later and *g*_s_ never decreased significantly. Although they represent average responses and summarize the performance of species with sometimes distinct behaviours, these results are consistent with previous demonstrations that drought sensitivity of C_4_ photosynthesis in grasses depends on metabolic rather than stomatal limitations (Ghannoum *et al*., 2003; Ripley *et al*., 2007, 2010). Ultimately, photosynthesis in both C_3_ and C_4_ grasses was limited by drought in our experiment, but our results suggest that the cause differed and show that significant effects on C_4_ photosynthesis occurred later. Consistent with our expectations, the greatest photosynthetic advantages for C_4_ grasses occurred during the development of drought in the warmest parts of the growing season.

We have previously shown that drought can narrow the gap in water-use efficiency between C_3_ and C_4_ grasses (Ripley *et al*., 2010; Taylor *et al*., 2011), which is large in well-watered, controlled conditions (e.g., Taylor *et al*., 2010 found that C_4_ *A*/*g*_s_ was double that of C_3_ grasses). Here we show that, under natural conditions, with a relatively diverse group of PACMAD species, the intrinsic water-use efficiency advantage to C_4_ species was much smaller when well watered (ca. 40% greater than C_3_). It was often difficult to distinguish C_3_ and C_4_ species based on differences in *A*/*g*_s_, which, outside of the wettest periods in the experiment were significant only during periods of intermediate drought. Assuming that water deficits were generally greater in the field environment than controlled growth conditions, this result is consistent with our previous finding that drought reduces the difference in intrinsic water-use efficiency between C_3_ and C_4_ grasses (Taylor *et al*., 2011), but implies that advantages may be regained as water availability continues to decline. The response of *A* to *g*_s_ is expected to saturate more quickly in C_3_ than C_4_ grasses (Osborne & Sack, 2012), and C_3_ species under well-watered conditions often operate above the point where increasing *g*_s_ results in diminishing returns for *A* (this study, data not shown). When faced with a need to reduce *g*_s_, the leaves of well-watered C_3_ plants initially face a relatively small penalty in *A*, and *A*/*g*_s_ increases, but C_4_ leaves retain a clear photosynthetic advantage at low *g*_s_ (Osborne & Sack, 2012). Importantly, we found that, when we accounted for leaf energy balance, the C_4_ advantage in *A*/*g*_s_ translated into significant differences in *A*/*E* only during periods of intermediate drought.

We were surprised to find that *g*_s_ was similar across the two photosynthetic types, even in watered plots. In previous comparisons of well-watered grasses from a diverse array of habitats, *g*_s_ was significantly higher among C_3_ grasses, though the full range of *g*_s_ observed across C_3_ and C_4_ species overlapped substantially (Taylor *et al*., 2010, 2011). However, we have previously observed similar *g*_s_ between C_3_ and C_4_ grasses in a subset of the species studied here (Frole, 2008; Ripley *et al*., 2010). We have also previously demonstrated that habitat water availability is important in determining stomatal trait differences among C_3_ and C_4_ grasses (Taylor *et al*., 2012), and it has been repeatedly shown that C_3_ and C_4_ lineages sort into distinct hydrological niches (Edwards & Still, 2008; Osborne & Freckleton, 2009; Edwards & Smith, 2010; Pau *et al*., 2012; Visser *et al*., 2014). It is possible that differential sensitivity to vapour pressure deficit between C_3_ and C_4_ leaves (Bunce, 1983; El-Sharkawy *et al*., 1985) contributed to the smaller difference in *g*_s_ values observed in these experiments. However, we suggest that similar *g*_s_ was observed among the species in this study because they were sampled from a restricted suite of habitats within a seasonally dry climate region. It follows that the smaller differences we observed in *A* and *A*/*g*_s_ may also depend on these factors. This interpretation reinforces the importance of plant water relations in structuring species assemblages, and leads to the prediction that differences in gas exchange traits between C_3_ and C_4_ grasses are likely to be more extreme among species from diverse habitats (Taylor *et al*., 2010, 2011, 2012).

The clear advantage for C_4_ grasses in midday gas exchange, particularly *A*, during the growing season implies a disadvantage to C_3_ grasses that might ultimately influence their local persistence. Of the deaths observed during the 2008–2009 growing season, the majority were among C_3_ plants, but they were not clearly associated with the watering treatment, a reminder that other factors may ultimately determine the local habitat preferences of these grasses (Visser *et al*., 2011). Seasonal differences in performance are one possibility: differences in leaf survival during winter have been demonstrated for the C_3_ and C_4_ subspecies of *A. semialata* when grown close to our field site (Ibrahim *et al*., 2008; Osborne *et al*., 2008), and a recent phylogenetic study investigating the grass flora of Hawaii demonstrated that the niche of C_3_ PACMAD species is associated with winter precipitation (Pau *et al*., 2012). Seasonal differences in productivity are also important in mixed C_3_/C_4_ grasslands (Ode *et al*., 1980; Still *et al*., 2003) and will no doubt be influenced by shifting patterns of precipitation and seasonality under global change. We found that performance of C_3_ and C_4_ grasses was most similar at the beginning and end of the growing season. A key question remaining to be tested, therefore, is whether performance and growth of our C_3_ species in the late autumn, winter and early spring offset the physiological advantages of their C_4_ relatives during the summer.

When the soil was wetter, leaf Ψ was less negative and ΔΨ smaller among the C_4_ than C_3_ species, consistent with our expectations (Taylor *et al*., 2010, 2011). During drought, we observed that ΔΨ decreased among C_3_ grasses and was maintained among C_4_ grasses. Because declines in ΔΨ were paired with decreasing *E* among C_3_ species, whereas *E* among C_4_ grasses increased with evaporative demand, we interpret the pattern of decreases in ΔΨ among C_3_ grasses as indicating greater vulnerability of their hydraulic systems to failure under drought. Smaller ΔΨ was associated with more negative Ψ_predawn_, not less negative Ψ_midday_. It is plausible that night-time rehydration under severe drought was insufficient to bring Ψ_predawn_ into equilibrium with soil Ψ in at least some of the C_3_ species (predawn disequilibrium; Donovan *et al*., 2001, 2003). Alternatively, increases of *K*_plant_, if genuine, may act to maintain *E*, hydration and physiological function in the face of increased evaporative demand (Jones, 1992). Changes in *K*_plant_ can be regulated by several physiological processes, including changes in tissue conductance due to aquaporin activity (Kaldenhoff *et al*., 2008); changes in water requirements of growing tissues (Boyer, 1985); or changes in mass allocation such that water supply via roots is enhanced relative to water demand from leaf area (Maseda & Fernández, 2006). Differences in hydraulic traits will have contributed to the overall differences in *K*_plant_ in this experiment, and one realistic possibility is that less negative Ψ and smaller ΔΨ in C_4_ grasses in this experiment was a result of better root system access to available soil water; we have previously observed that the C_4_ lineages in this experiment have higher root mass ratios than the C_3_ lineages (Taylor *et al*., 2010). However, it is important to note that, although *K*_plant_ tended to be lower among C_3_ species, it was not always so: although suggestive, our evidence is not sufficient to claim that a clear difference in *K*_plant_ between C_3_ and C_4_ species was the principal driver for C_4_ performance advantages. Nonetheless, our results highlight a need to address mechanistic questions about the integration of hydraulic and photosynthetic performance among grasses, ideally in field experiments and especially under drought.

The characterization of ecophysiological traits associated with C_4_ photosynthesis is vital for understanding the natural diversity and ecological success of C_4_ species, and the differential impacts of global change on C_3_ and C_4_ species. The comparisons reported here, using four lineages of PACMAD grasses sampled from the same regional species pool and grown under natural climatic conditions, are a crucial complement to previous experiments that were pot-based, carried out in controlled environments, or completed using a less diverse panel of species. Our experimental manipulation of water availability influenced contrasts in leaf physiology during a growing season and we found that C_4_ photosynthetic advantages were maintained when a diverse panel of grass species were exposed to natural water shortages. Under mild drought, the C_4_ advantage in *A* was increased, as C_3_ leaves faced stomatal limitation of photosynthesis associated with earlier decreases in Ψ. We show that, under native climatic conditions in a location where both C_3_ and C_4_ PACMAD grasses are naturally abundant, water availability plays a crucial role in determining the magnitude of differences in physiological performance associated with photosynthetic type. Importantly, our experimental evidence supports a need to rigorously examine the proposition that advantages of C_4_ photosynthesis in dry environments are significantly modified by dynamic responses of the stomata and hydraulic system to drought (Osborne & Sack, 2012). Understanding the interplay between C_4_ photosynthesis and hydraulics will be crucial as we aim to better understand the response of plant communities to global change, including the question of why the distribution of C_4_ PACMAD grasses is so strongly linked with water availability (Edwards & Smith, 2010; Pau *et al*., 2012).

## Supporting Information

Data S1 Equations used for estimation of reference crop evapotranspiration (ET_0_).

Data S2 Model for estimation of leaf-level transpiration (*E*).

Data S3 Mean values for microclimate used in modelling of leaf-level transpiration (*E*).

Data S4 Tabulated values for physiological traits determined in the field, by species, month of measurement and treatment.

Data S5 Plotted species means used for analysis.
