# Supplementary material for: Physiological advantages of C4 grasses in the field: a comparative experiment demonstrating the importance of drought
Source: Glob Chang Biol. 2014 Mar 28;20(6):1992–2003. doi: 10.1111/gcb.12498 (PMC4237462; doi:10.1111/gcb.12498)
Supplement: Data S1 — Equations used for estimation of reference crop evapotranspiration (ET0). [file gcb0020-1992-SD1.pdf]

Taylor *et al*. supporting information

S1) Equations used for estimation of reference crop evapotranspiration (ET_0)

PET (potential evapotranspiration, mm d^{-1}) was derived from daily mean values calculated from weather station data. With the exception of the approximation made for incoming solar radiation (R_s), which is based on information on p. 22 of Jones (1992), the methods follow recommendations made by Allen *et al*. (1998) for the calculation of reference crop evapotranspiration (ET_0). These methods assume a 'hypothetical reference crop with an assumed crop height of 0.12 m, a fixed surface resistance of 70 s m^{-1} and an albedo of 0.23 reference surface' (Allen *et al*., 1998) and meterological measurements made at 2 m height above the ground surface. The result is that aerodynamic resistance r_a (s m^{-1}) has the approximation 208/w, where w = windspeed (m s^{-1}).

$$ET_0 = \frac{0.408\varepsilon R_{net} + \gamma \frac{900}{T_{air} + 273} w\delta e}{\varepsilon + \gamma(1 + 0.34w)} \quad eqn. S1.1$$

T_{air} is daily mean air temperature (°C), the average of daily maximum (T_{max}, °C) and minimum air temperatures (T_{min}, °C):

$$T_{air} = \frac{T_{min} + T_{max}}{2} \quad eqn. S1.2$$

w is daily mean windspeed at 2 m (m s^{-1}), calculated similarly to daily mean temperature.

$$\delta e = e_s - e_a \quad eqn. S1.3$$

e_s is daily mean saturation vapour pressure (kPa), the average of saturation vapour pressures at daily maximum (T_{max}, °C) and minimum air temperatures (T_{min}, °C):

$$e_s = \frac{e_{s,Tmin} + e_{s,Tmax}}{2} \quad eqn. S1.4$$

$$e_{s,T} = 0.6108 exp\left(\frac{17.27T}{T+237.3}\right) \quad eqn. S1.5$$

e_a is actual vapour pressure (kPa), calculated based on daily maximum (RH_{max}, %) and minimum (RH_{min}, %) relative humidities:

$$e_a = \frac{e_{s,Tmin}\left(\frac{RH_{max}}{100}\right) + e_{s,Tmax}\left(\frac{RH_{min}}{100}\right)}{2} \quad eqn. S1.6$$

ε is the slope of vapour pressure curve (kPa °C^{-1}):

$$\varepsilon = \frac{4098\left(0.6108 exp\left(\frac{17.27T_{air}}{T_{air} + 237.3}\right)\right)}{(T_{air} + 237.3)^2} \quad eqn. S1.7$$

γ is the psychrometric constant (kPa °C^{-1}):

$$\gamma = 0.665 \times 10^{-3} P \quad eqn. S1.8$$

P is air pressure (kPa):

$$P = 101.3\left(\frac{293 - 0.0065z}{293}\right)^{5.26} \quad eqn. S1.9$$

z = site elevation (630 m)

R_{net} is net radiation (MJ m^{-2} d^{-1}), calculated as the difference between net solar radiation (R_{ns}, MJ m^{-2} d^{-1}) and net longwave radiation (R_{nl}, MJ m^{-2} d^{-1}):

$$R_n = R_{ns} - R_{nl} \quad eqn. S1.10$$

$R_{ns} = (1 - \alpha)R_s \quad eqn. S1.11$

α is the albedo of the crop, 0.23 for the reference crop

R_s is the solar radiation (MJ m^{-2} d^{-1}), approximated as shortwave irradiance, calculated from integrated daily PPFD (µmol m^{-2} d^{-1}) on the basis of information presented in Jones (1992; Table 2.2):

$$R_s = \frac{PPFD}{457 \times 200 \ \times 10^6} \quad eqn. S1.12$$

$$R_{nl} = \sigma\left(\frac{T_{min,K}{}^4 + T_{max,K}{}^4}{2}\right)\left(0.34 - 0.14\sqrt{\mathrm{e_a}}\right)\left(1.35\frac{\mathrm{R_s}}{\mathrm{R_{so}}} - 0.35\right) \quad eqn. S1.13$$

σ is the Stefan-Boltzmann constant: 4.903 10^{-9} MJ K^{-4} m^{-2} d^{-1}

$T_{min,K}$ and $T_{max,K}$ are the daily minimum and maximum temperatures (K), respectively:

$$T_K = T_{°\mathrm{C}} + 273.16 \quad eqn. S1.14$$

R_{so} is the clear sky radiation (MJ m^{-2} d^{-1}):

$$R_{so} = (a_s + b_s)R_a \quad eqn. S1.15$$

a_s and b_s are Angstrom constants: 0.25 and 0.5 respectively

R_a is the extraterrestrial radiation (MJ m^{-2} d^{-1}):

$$R_a = \frac{24(60)}{\pi} G_{sc} d_r\left(\omega_s \sin(\varphi)\sin(\delta) + \cos(\varphi)\cos(\delta)\sin(\omega_s)\right) \quad eqn. S1.16$$

π is 3.141593

G_{sc} is the solar constant: 0.082 MJ m^{-2} min^{-1}

d_r is the inverse relative distance Earth-Sun:

$$d_r = 1 + 0.033cos\left(\frac{2\pi}{365}J\right) \quad eqn. S1.17$$

J is the Julian day

ω_s is the sunset hour angle (rad):

$$\omega_s = arccos\big(-\tan(\varphi)\tan(\delta)\big)$$

φ is the latitude of the experimental site in this study: −0.5784021 rad

δ is solar declination (rad):

$$\delta = 0.409sin\left(\frac{2\pi}{365}J - 1.39\right) \quad eqn. S1.18$$

References

Allen RG, Pereira LS, Raes D, Smith M (1998) *Crop evapotranspiration - Guidelines for computing crop water requirements - FAO Irrigation and drainage paper 56.* Rome, FAO - Food and Agriculture Organization of the United Nations.

Jones HG (1992) *Plants and microclimate: a quantitative approach to environmental plant physiology*. Cambridge, United Kingdom, Cambridge University Press.
