# Supplementary material for: Physiological advantages of C4 grasses in the field: a comparative experiment demonstrating the importance of drought
Source: Glob Chang Biol. 2014 Mar 28;20(6):1992–2003. doi: 10.1111/gcb.12498 (PMC4237462; doi:10.1111/gcb.12498)
Supplement: Data S2 — Model for estimation of leaf-level transpiration (E). [file gcb0020-1992-SD2.pdf]

Taylor *et al*. supporting information

S2) Model for estimation of leaf-level transpiration (*E*)

The Penman-Monteith equation was combined with an iterative approach to model the leaf energy balance for a horizontal leaf suspended over a lawn. The model was parameterised using mean values for climate variables (Supplementary S3) and g_s (Supplementary S4) from each measurement period during the growing season. Methods below follow Jones (1992) unless noted.

The simplifying assumption was made that the leaves showed negligible loss of heat to sinks other than the air, thus we used the following modified version of the Penman-Monteith equation (Penman, 1948; Monteith, 1965):

$$\lambda E = \frac{\varepsilon R_{NET} + \rho_a c_p g_H \delta e}{\varepsilon + \left(\gamma \frac{g_H}{g_w}\right)} \quad eqn. S2.1$$

λE, latent heat flux (J m^{-2} s^{-1})

$E = \frac{\lambda E}{18 \times \lambda_{TairC}}$ (mmol m^{-2} s^{-1}) $\quad eqn. S2.2$

λ_{TairC}, latent heat of vaporisation ≤ 2.513 MJ kg^{-1}

$\lambda_{TairC} = 1000(3.1512 \times 10^{-3} - 2.38 \times 10^{-6}(T_{airC} + 273))$ (Friend, 1995) $\quad eqn. S2.3$

T_{airC}, mean daily maximum of air temperature at 0.50 m during measurement period (°C)

continues overleaf...

ε, temperature dependent slope of the vapour pressure-temperature relationship (Pa K^{-1})

$$\varepsilon = \frac{\left(\mathrm{e}_{\mathrm{s,TairC}} - \mathrm{e}_{\mathrm{s,TairC-0.1}}\right)}{\mathrm{T}_{\mathrm{airC}} - (\mathrm{T}_{\mathrm{airC}} - 0.1)} \quad eqn.S2.4$$

$$\mathrm{e}_{\mathrm{s,TairC}} = 613.75\mathrm{exp}\left(\frac{17.502\mathrm{T}_{\mathrm{airC}}}{240.97 + \mathrm{T}_{\mathrm{airC}}}\right) \quad eqn.S2.5$$

R_{NET}, instantaneous net radiation (J m^{-2} s^{-1}): derived from a model of leaf energy balance for a horizontal leaf suspended over a lawn

$$R_{NET} = \alpha_{s,leaf}\left(I_s + I_s\rho_{s,lawn}\right) + I_{ld} + \sigma T_{lawn}{}^4 - 2\sigma T_{leaf}{}^4 \quad eqn.S2.6$$

The temperatures (K), of the lawn above which the leaf was considered to be suspended, T_{lawn}, and of the leaf, T_{leaf}, were determined by iteration. Between each round of simulation T_{lawn} was set to match T_{leaf}. The model was considered to have converged once change in T_{leaf} between rounds of simulation was <0.1 °C.

$\alpha_{s,leaf}$, leaf absorption coefficient for shortwave radiation = 0.5

$\rho_{s,lawn}$, reflection coefficient for lawn = 0.23

σ, Stefan-Boltzmann constant = 5.6703×10^{-8} J m^{-2} K^{-4} s^{-1}

I_s, shortwave irradiance (J m^{-2} s^{-1})

$I_s = 200/457 \times PPFD_{max}$ (Table 2.2 of Jones, 1992) $\quad eqn.S2.7$

$PPFD_{max}$, maximum daily PPFD (μmol m^{-2} s^{-1})

continues overleaf...

I_{ld}, downward irradiance in the longwave assuming a clear sky

$$I_{ld} = \sigma(T_{airK} - 20)^4 \quad eqn.S2.8$$

T_{airK}, air temperature (K)

$$T_{airK} = T_{airC} + 273.16 \quad eqn.S2.9$$

ρ_a, temperature dependent density of dry air ≤ 1.317 kg m^{-3}

$$\rho_a = \frac{P}{R_{air}(T_{airC} + 273)} \quad eqn.S2.10$$

P, air pressure (Pa)

R_{air}, specific gas constant for air (J K^{-1} kg^{-1})

$$R_{air} = \frac{R}{M_{air}} \quad eqn.S2.11$$

R, gas constant = 8.3144 J K^{-1} mol^{-1}

M_{air}, molecular weight of air = 28.964×10^{-3} kg mol^{-1}

c_p, specific heat capacity of water = 1011 J kg^{-1} K^{-1}

continues overleaf...

g_H, boundary layer conductance (m s^{-1})

$$g_H = 6.62 \times 10^{-3}(w_{0.5}/d)^{0.5} \quad eqn.S2.12$$

$w_{0.5}$, mean windspeed at 0.5 m during measurement period (m s^{-1})

$$w_{0.5} = \frac{u^*}{0.4}\log\frac{0.5(1-0.65)}{z_m} \text{ (Campbell \& Norman,1998; chapter 5)} \quad eqn.S2.13$$

$$u^* = 0.4\left[\frac{w}{\log\left(\frac{2-d_0}{z_m}\right)}\right] \quad eqn.S2.14$$

w, mean windspeed at 2 m during measurement period (m s^{-1})

d_0, zero plane displacement (m)

$$d_0 = 0.65h \quad eqn.S2.15$$

h, height of lawn surrounding plots = 0.02 m

z_m, momentum roughness parameter (m)

$$z_m = 0.1h \quad eqn.S2.16$$

continues overleaf...

d, average leaf characteristic dimension (m): average of maximum and minimum widths reported by Gibbs Russell *et al.* (1990); where a minimum was not reported it was assumed to be 0.5 mm.

Species	Leaf width (mm)
Themeda triandra	4.5
Heteropogon contortus	5.4
Hypparhenia hirta	4.25
Aristida diffiusa	1.25
Aristida junciformis	1.75
Aristida congests	0.75
Panicum ecklonii	5.5
Alloteropsis eckloniana	7.5
Panicum aequinerve	6.5
Pentaschistis curvifolia	2.25
Merxmuellera disticha	2
Karoochloa curva	1.25

δe, vapour pressure deficit (Pa)

$$\delta e = e_{s,TairC} - e_a \quad eqn. S2.17$$

e_a, ambient vapour pressure (Pa)

$$e_a = \frac{RH}{100} e_{s,TairC} \quad eqn. S2.18$$

RH, mean relative humidity (%)

γ, temperature and air-pressure dependent value of the psychrometric constant (Pa K^{-1})

$$\gamma = \frac{Pc_p}{0.622(\lambda_{TairC} \times 10^6)} \quad eqn. S2.19$$

continues overleaf...

g_W, leaf conductance to water vapour (m s^{-1})

$$g_W = \frac{1}{\frac{1}{g_H} + \frac{1}{g_s}} \quad eqn. S2.20$$

g_s, mean stomatal conductance during measurement period (m s^{-1}), for units conversion see Appendix 3 of Jones (1992)

References

Campbell GS, Norman JM (1998) *Introduction to environmental biophysics*. Springer Science + Business Media, New York.

Friend AD (1995) PGEN: An integrated model of leaf photosynthesis, transpiration, and conductance. *Ecological Modelling*, **77**, 233-255.

Gibbs Russell GE, Watson L, Koekmoer L, Smook L, Barker NP, Anderson HM, Dallwitz MJ (1990) *Grasses of Southern Africa*. Pretoria, National Botanical Gardens.

Jones HG (1992) *Plants and microclimate: a quantitative approach to environmental plant physiology*. Cambridge, United Kingdom, Cambridge University Press.

Penman HL (1948) Natural evaporation from open water, bare soil and grass. *Proceedings of the Royal Society A*, **193**,120-145.

Monteith JL (1965) Evaporation and environment. *Symposia of the Society for Experimental Biology*, **19**, 205-234.
