# Supplementary material for: Physiological advantages of C4 grasses in the field: a comparative experiment demonstrating the importance of drought
Source: Glob Chang Biol. 2014 Mar 28;20(6):1992–2003. doi: 10.1111/gcb.12498 (PMC4237462; doi:10.1111/gcb.12498)
Supplement: Data S3 — Mean values for microclimate used in modelling of leaf-level transpiration (E). [file gcb0020-1992-SD3.pdf]

Taylor *et al*. Supplementary materials

S3) Mean values for microclimate used in modelling of leaf-level transpiration (*E*)

Values are means, for each sampling period, of daily values for each parameter. Windspeed was recorded at 2 m, PPFD was recorded at 1.5 m, relative humidity and temperature were recorded at 0.5 m

Period	Minimum windspeed ($m\ s^{-1}$)	Maximum windspeed ($m\ s^{-1}$)
November 17th-28th 2008	0.58	5.52
December 12th-31st 2008	1.08	6.49
January 18th-27th 2009	0.65	6.62
February 16th-28th 2009	0.52	5.07
March 15th-31st 2009	1.03	5.99
April 15th-26th 2009	0.83	5.63

Period	Maximum Relative Humidity (%)	Minimum Relative Humidity (%)	Maximum Temperature (°C)	Minimum Temperature (°C)
November 17th-28th 2008	91.9	56.5	25.5	13.0
December 12th-31st 2008	88.2	55.0	28.0	13.9
January 18th-27th 2009	89.4	47.6	30.4	14.0
February 16th-28th 2009	91.6	65.7	26.9	14.8
March 15th-31st 2009	91.3	47.7	24.2	11.1
April 15th-26th 2009	97.1	47.7	23.6	12.2

Period	Maximum PPFD ($\mu mol\ m^{-2}\ s^{-1}$)
November 17th-28th 2008	1843
December 12th-31st 2008	1956
January 18th-27th 2009	2006
February 16th-28th 2009	1732
March 15th-31st 2009	1688
April 15th-26th 2009	1357
