# Supplementary material for: Physiological advantages of C4 grasses in the field: a comparative experiment demonstrating the importance of drought
Source: Glob Chang Biol. 2014 Mar 28;20(6):1992–2003. doi: 10.1111/gcb.12498 (PMC4237462; doi:10.1111/gcb.12498)
Supplement: Data S4 — Tabulated values for physiological traits determined in the field, by species, month of measurement and treatment. [file gcb0020-1992-SD4.pdf]

Taylor *et al*. supporting information

S4) Tabulated values for physiological traits determined in the field, by species, month of measurement and treatment

The mean values on the following four pages were calculated from raw data, using R version 3.0.1 (R Core Team, 2013). Mean values based on ≤ 2 replicates were eliminated from the dataset, as were species means that were not paired across treatments in any given month.

Species codes:

Hc = *Heteropogon contortus* (C_4, Andropogoneae)
Hh = *Hyparrhenia hirta* (C_4, Andropogoneae)
Tt = *Themeda triandra* (C_4, Andropogoneae)

As = *Alloteropsis semialata ss. eckloniana* (C_3, Paniceae)
Pa = *Panicum aequinerve* (C_3, Paniceae)
Pe = *Panicum ecklonii* (C_3, Paniceae)

Ac = *Aristida congesta ss. barbicollis* (C_4, Aristidoideae)
Ad = *Aristida diffusa ss. diffusa* (C_4, Aristidoideae)
Aj = *Aristida junciformis ss. junciformis* (C_4, Aristidoideae)

Kc = *Karoochloa curva* (C_3, Danthonioideae)
Md = *Merxmuellera disticha* (C_3, Danthonioideae)
Pc = *Pentaschistis curvifolia* (C_3, Danthonioideae)

Treatment codes:

n = natural rainfall
w = supplemental watering

N = sample sizes

Missing values are indicated by hyphens

References

R Core Team (2013) R: A language and environment for statistical computing. R Foundation for Statistical Computing, Vienna, Austria.

Andropogoneae (C_4)

Species[1]	Month	Treatment[2]	Net CO_2 assimilation rate (μmol CO_2 m^{-2} s^{-1})	N	Stomatal conductance (mol m^{-2} s^{-1})	N	Midday leaf water potential (MPa)	N	Pre-dawn leaf water potential (MPa)	N
Hc	Nov-08	n	7.95	7	0.108	7	-1.70	7	-0.38	6
Hc	Dec-08	n	11.23	5	0.142	5	-1.60	4	-0.30	4
Hc	Jan-09	n	6.79	5	0.099	5	-2.44	5	-0.69	4
Hc	Feb-09	n	15.80	6	0.131	6	-1.57	6	-0.21	5
Hc	Mar-09	n	8.56	6	0.078	6	-1.88	6	-0.42	6
Hc	Apr-09	n	8.65	7	0.088	7	-2.06	7	-1.00	7
Hc	Nov-08	w	7.05	8	0.100	8	-1.89	8	-0.36	7
Hc	Dec-08	w	10.98	5	0.137	5	-1.68	4	-0.28	4
Hc	Jan-09	w	12.29	6	0.127	6	-1.58	6	-0.51	5
Hc	Feb-09	w	15.92	7	0.138	7	-1.39	7	-0.17	6
Hc	Mar-09	w	16.09	7	0.120	7	-1.57	7	-0.30	7
Hc	Apr-09	w	11.17	8	0.112	8	-1.73	8	-0.88	8
Hh	Nov-08	n	20.17	8	0.151	8	-1.19	6	-0.29	6
Hh	Dec-08	n	18.32	5	0.135	5	-1.23	4	-0.19	4
Hh	Jan-09	n	11.36	6	0.081	6	-1.98	6	-1.16	5
Hh	Feb-09	n	18.24	7	0.154	7	-1.24	7	-0.22	6
Hh	Mar-09	n	14.84	7	0.096	7	-1.67	7	-0.34	7
Hh	Apr-09	n	10.47	8	0.091	8	-2.48	8	-1.35	8
Hh	Nov-08	w	19.01	8	0.137	8	-1.26	6	-0.35	6
Hh	Dec-08	w	20.32	6	0.153	6	-1.21	4	-0.17	3
Hh	Jan-09	w	14.53	6	0.112	6	-1.48	6	-0.58	5
Hh	Feb-09	w	13.96	7	0.105	7	-1.20	7	-0.18	6
Hh	Mar-09	w	16.49	7	0.116	7	-1.43	7	-0.36	7
Hh	Apr-09	w	14.75	8	0.119	8	-1.76	8	-0.91	8
Tt	Nov-08	n	-	-	-	-	-	-	-	-
Tt	Dec-08	n	-	-	-	-	-	-	-	-
Tt	Jan-09	n	10.21	5	0.093	5	-1.85	4	-0.49	4
Tt	Feb-09	n	5.07	7	0.072	7	-2.90	7	-1.88	6
Tt	Mar-09	n	12.06	7	0.095	7	-1.67	7	-0.32	6
Tt	Apr-09	n	9.78	7	0.088	7	-2.21	7	-0.54	7
Tt	Nov-08	w	5.55	8	0.076	8	-2.42	8	-1.33	8
Tt	Dec-08	w	12.59	6	0.105	6	-1.92	3	-0.20	3
Tt	Jan-09	w	14.17	6	0.106	6	-2.05	6	-0.49	5
Tt	Feb-09	w	12.57	7	0.110	7	-1.67	7	-0.24	6
Tt	Mar-09	w	13.15	6	0.100	6	-2.05	6	-0.43	6
Tt	Apr-09	w	10.47	7	0.098	7	-1.96	7	-1.08	7

Continued overleaf...

Paniceae (C_3)

Species[1]	Month	Treatment[2]	Net CO_2 assimilation rate (μmol CO_2 m^{-2} s^{-1})	N	Stomatal conductance (mol m^{-2} s^{-1})	N	Midday leaf water potential (MPa)	N	Pre-dawn leaf water potential (MPa)	N
As	Nov-08	n	9.10	8	0.114	8	-1.58	8	-0.39	7
As	Dec-08	n	6.48	5	0.054	5	-1.53	4	-0.38	4
As	Jan-09	n	1.76	6	0.027	6	-2.35	6	-1.19	5
As	Feb-09	n	11.55	7	0.156	7	-1.54	7	-0.23	6
As	Mar-09	n	5.42	7	0.050	7	-1.71	7	-0.56	7
As	Apr-09	n	4.47	7	0.038	7	-2.07	7	-1.25	7
As	Nov-08	w	10.41	7	0.116	7	-1.49	7	-0.53	6
As	Dec-08	w	10.26	6	0.094	6	-1.33	4	-0.24	4
As	Jan-09	w	6.36	6	0.082	6	-1.70	6	-0.58	5
As	Feb-09	w	10.77	7	0.147	7	-1.37	7	-0.21	6
As	Mar-09	w	11.11	6	0.116	6	-1.55	6	-0.37	6
As	Apr-09	w	8.94	7	0.105	7	-1.66	7	-1.09	7
Pa	Nov-08	n	12.92	8	0.131	8	-1.40	3	-0.43	3
Pa	Dec-08	n	3.80	5	0.032	5	-1.43	4	-0.41	4
Pa	Jan-09	n	-0.03	6	0.032	6	-3.20	5	-3.00	4
Pa	Feb-09	n	14.91	7	0.215	7	-1.61	7	-0.22	6
Pa	Mar-09	n	9.51	7	0.071	7	-2.09	7	-0.77	7
Pa	Apr-09	n	7.55	8	0.066	8	-2.55	8	-1.71	7
Pa	Nov-08	w	15.08	8	0.171	8	-1.35	2	-0.30	2
Pa	Dec-08	w	11.46	5	0.120	5	-1.64	4	-0.23	4
Pa	Jan-09	w	7.25	6	0.067	6	-1.95	6	-0.62	5
Pa	Feb-09	w	18.56	7	0.284	7	-1.47	7	-0.19	6
Pa	Mar-09	w	14.35	7	0.160	7	-1.77	7	-0.41	7
Pa	Apr-09	w	17.46	8	0.223	8	-2.20	8	-0.99	8
Pe	Nov-08	n	7.20	6	0.099	6	-1.85	3	-0.98	2
Pe	Dec-08	n	-	-	-	-	-	-	-	-
Pe	Jan-09	n	-	-	-	-	-	-	-	-
Pe	Feb-09	n	11.51	4	0.147	4	-1.78	4	-0.43	3
Pe	Mar-09	n	8.68	3	0.094	3	-1.60	2	-0.85	2
Pe	Apr-09	n	4.39	3	0.067	3	-2.28	2	-1.05	2
Pe	Nov-08	w	10.34	7	0.123	7	-1.78	3	-0.43	2
Pe	Dec-08	w	-	-	-	-	-	-	-	-
Pe	Jan-09	w	-	-	-	-	-	-	-	-
Pe	Feb-09	w	12.00	4	0.182	4	-1.63	3	-0.23	3
Pe	Mar-09	w	8.89	4	0.093	4	-1.73	3	-0.30	3
Pe	Apr-09	w	15.50	4	0.177	4	-2.43	3	-1.15	3

Continued overleaf...

***Aristida* spp. (C_4)**

Species[1]	Month	Treatment[2]	Net CO_2 assimilation rate ($\mu mol\ CO_2\ m^{-2}\ s^{-1}$)	N	Stomatal conductance ($mol\ m^{-2}\ s^{-1}$)	N	Midday leaf water potential (MPa)	N	Pre-dawn leaf water potential (MPa)	N
Ac	Nov-08	n	-	-	-	-	-	-	-	-
Ac	Dec-08	n	-	-	-	-	-	-	-	-
Ac	Jan-09	n	-	-	-	-	-	-	-	-
Ac	Feb-09	n	13.11	3	0.135	3	-1.37	3	-0.33	3
Ac	Mar-09	n	13.88	4	0.114	4	-1.90	4	-0.34	4
Ac	Apr-09	n	-	-	-	-	-	-	-	-
Ac	Nov-08	w	-	-	-	-	-	-	-	-
Ac	Dec-08	w	-	-	-	-	-	-	-	-
Ac	Jan-09	w	-	-	-	-	-	-	-	-
Ac	Feb-09	w	16.66	6	0.145	6	-1.52	6	-0.30	5
Ac	Mar-09	w	16.97	6	0.169	6	-1.73	6	-0.49	6
Ac	Apr-09	w	-	-	-	-	-	-	-	-
Ad	Nov-08	n	14.87	5	0.172	5	-1.67	5	-0.45	5
Ad	Dec-08	n	19.58	3	0.148	3	-1.52	3	-0.28	3
Ad	Jan-09	n	4.91	6	0.196	6	-2.60	6	-1.70	5
Ad	Feb-09	n	21.93	6	0.207	6	-1.42	6	-0.30	5
Ad	Mar-09	n	21.33	6	0.150	6	-1.88	6	-0.43	6
Ad	Apr-09	n	8.82	7	0.158	7	-2.04	7	-0.93	7
Ad	Nov-08	w	13.53	6	0.194	6	-1.75	6	-0.80	5
Ad	Dec-08	w	15.25	6	0.187	6	-1.58	4	-0.31	4
Ad	Jan-09	w	13.50	6	0.170	6	-1.77	6	-0.68	5
Ad	Feb-09	w	13.69	7	0.214	7	-1.44	7	-0.28	6
Ad	Mar-09	w	17.45	7	0.165	7	-1.69	7	-0.42	7
Ad	Apr-09	w	14.58	8	0.194	8	-1.91	8	-0.80	8
Aj	Nov-08	n	3.99	8	0.136	8	-1.60	8	-0.31	7
Aj	Dec-08	n	10.56	5	0.075	5	-1.70	4	-0.16	4
Aj	Jan-09	n	1.91	6	0.072	6	-2.80	6	-1.52	5
Aj	Feb-09	n	5.14	6	0.167	6	-1.50	6	-0.24	5
Aj	Mar-09	n	9.20	7	0.233	7	-1.91	7	-0.33	7
Aj	Apr-09	n	5.46	8	0.245	8	-2.20	8	-1.15	8
Aj	Nov-08	w	7.82	8	0.131	8	-1.73	8	-0.29	7
Aj	Dec-08	w	11.24	6	0.097	6	-1.73	4	-0.18	4
Aj	Jan-09	w	4.17	6	0.136	6	-1.70	6	-0.45	5
Aj	Feb-09	w	7.63	6	0.153	6	-1.47	6	-0.17	5
Aj	Mar-09	w	10.08	7	0.141	7	-1.83	7	-0.39	7
Aj	Apr-09	w	11.63	8	0.178	8	-1.65	8	-0.76	8

Continued overleaf...

Danthonioideae (C_3)

Species[1]	Month	Treatment[2]	Net CO_2 assimilation rate (μmol CO_2 m^{-2} s^{-1})	N	Stomatal conductance (mol m^{-2} s^{-1})	N	Midday leaf water potential (MPa)	N	Pre-dawn leaf water potential (MPa)	N
Kc	Nov-08	n	5.89	8	0.107	8	-1.89	4	-0.73	4
Kc	Dec-08	n	4.46	3	0.116	3	-1.75	2	-0.75	2
Kc	Jan-09	n	0.07	4	0.140	4	-3.00	4	-1.50	4
Kc	Feb-09	n	10.63	6	0.196	6	-1.62	6	-0.40	5
Kc	Mar-09	n	1.65	5	0.085	5	-2.24	5	-0.59	5
Kc	Apr-09	n	2.64	6	0.108	6	-2.73	6	-1.53	5
Kc	Nov-08	w	3.85	5	0.089	5	-2.03	5	-0.76	4
Kc	Dec-08	w	3.51	3	0.129	3	-2.70	3	-0.47	3
Kc	Jan-09	w	3.33	4	0.150	4	-2.23	4	-0.88	3
Kc	Feb-09	w	7.88	4	0.193	4	-1.75	4	-0.36	4
Kc	Mar-09	w	5.83	3	0.130	3	-2.33	3	-0.50	3
Kc	Apr-09	w	11.60	3	0.161	3	-2.37	3	-1.08	3
Md	Nov-08	n	6.94	7	0.138	7	-2.75	6	-0.48	6
Md	Dec-08	n	9.19	5	0.138	5	-3.00	4	-0.54	4
Md	Jan-09	n	1.78	6	0.055	6	-2.95	6	-1.73	4
Md	Feb-09	n	10.76	4	0.185	4	-2.08	4	-0.35	4
Md	Mar-09	n	3.71	7	0.087	7	-2.54	7	-0.57	7
Md	Apr-09	n	6.11	8	0.102	8	-2.70	8	-1.26	8
Md	Nov-08	w	10.08	8	0.136	8	-2.42	7	-0.70	7
Md	Dec-08	w	9.23	5	0.132	5	-2.28	4	-0.30	4
Md	Jan-09	w	5.73	6	0.137	6	-2.45	6	-0.77	5
Md	Feb-09	w	5.73	5	0.170	5	-1.90	5	-0.39	5
Md	Mar-09	w	7.15	7	0.117	7	-2.31	7	-0.66	7
Md	Apr-09	w	7.56	8	0.127	8	-2.51	8	-1.16	8
Pc	Nov-08	n	5.46	7	0.073	7	-2.13	6	-0.47	5
Pc	Dec-08	n	7.86	3	0.095	3	-1.75	3	-0.33	3
Pc	Jan-09	n	-	-	-	-	-	-	-	-
Pc	Feb-09	n	9.80	4	0.121	4	-2.05	4	-0.35	3
Pc	Mar-09	n	0.91	4	0.086	4	-2.58	4	-1.26	4
Pc	Apr-09	n	3.23	6	0.059	6	-2.38	6	-1.27	6
Pc	Nov-08	w	10.48	6	0.137	6	-2.23	6	-0.53	6
Pc	Dec-08	w	10.41	5	0.133	5	-2.04	4	-0.23	4
Pc	Jan-09	w	-	-	-	-	-	-	-	-
Pc	Feb-09	w	7.44	7	0.129	7	-1.96	7	-0.26	6
Pc	Mar-09	w	6.26	6	0.092	6	-2.27	6	-0.53	6
Pc	Apr-09	w	6.52	6	0.100	6	-2.14	5	-0.94	5
