# Supplementary material for: Physiological advantages of C4 grasses in the field: a comparative experiment demonstrating the importance of drought
Source: Glob Chang Biol. 2014 Mar 28;20(6):1992–2003. doi: 10.1111/gcb.12498 (PMC4237462; doi:10.1111/gcb.12498)
Supplement: Data S5 — Plotted species means used for analysis. [file gcb0020-1992-SD5.pdf]

Taylor *et al*. supporting information

S5) Plotted species means used for analysis

The mean values plotted by species, month and treatment on the following five pages were calculated from raw data, using R version 3.0.1 (R Core Team, 2013). Mean values based on ≤ 2 replicates were eliminated from the dataset, as were species means that were not paired across treatments in any given month. Error bars, where given, are SEM; where error bars are missing this is because values were calculated from species means, which represent a single value rather than measurements replicated across several plants.

Species codes:

Hc = *Heteropogon contortus* (C_4, Andropogoneae)
Hh = *Hyparrhenia hirta* (C_4, Andropogoneae)
Tt = *Themeda triandra* (C_4, Andropogoneae)

As = *Alloteropsis semialata ss. eckloniana* (C_3, Paniceae)
Pa = *Panicum aequinerve* (C_3, Paniceae)
Pe = *Panicum ecklonii* (C_3, Paniceae)

Ac = *Aristida congesta ss. barbicollis* (C_4, Aristidoideae)
Ad = *Aristida diffusa ss. diffusa* (C_4, Aristidoideae)
Aj = *Aristida junciformis ss. junciformis* (C_4, Aristidoideae)

Kc = *Karoochloa curva* (C_3, Danthonioideae)
Md = *Merxmuellera disticha* (C_3, Danthonioideae)
Pc = *Pentaschistis curvifolia* (C_3, Danthonioideae)

References

R Core Team (2013) R: A language and environment for statistical computing. R Foundation for Statistical Computing, Vienna, Austria.

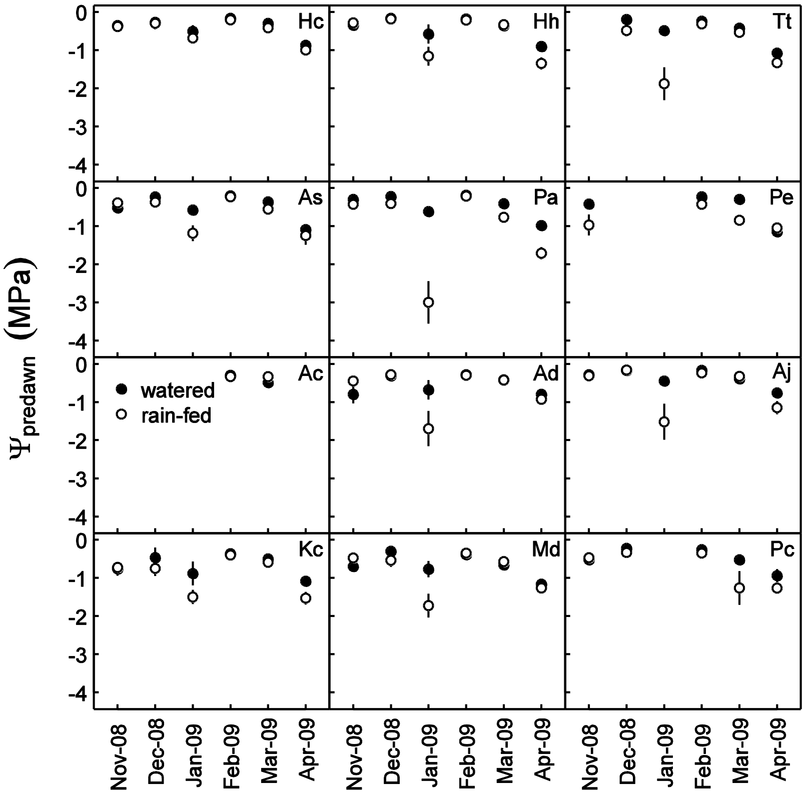

Ψpredawn (MPa)
Hc
Hh
Tt
As
Pa
Pe
Ac
Ad
Aj
Kc
Md
Pc
watered
rain-fed
0
-1
-2
-3
-4
Nov-08
Dec-08
Jan-09
Feb-09
Mar-09
Apr-09

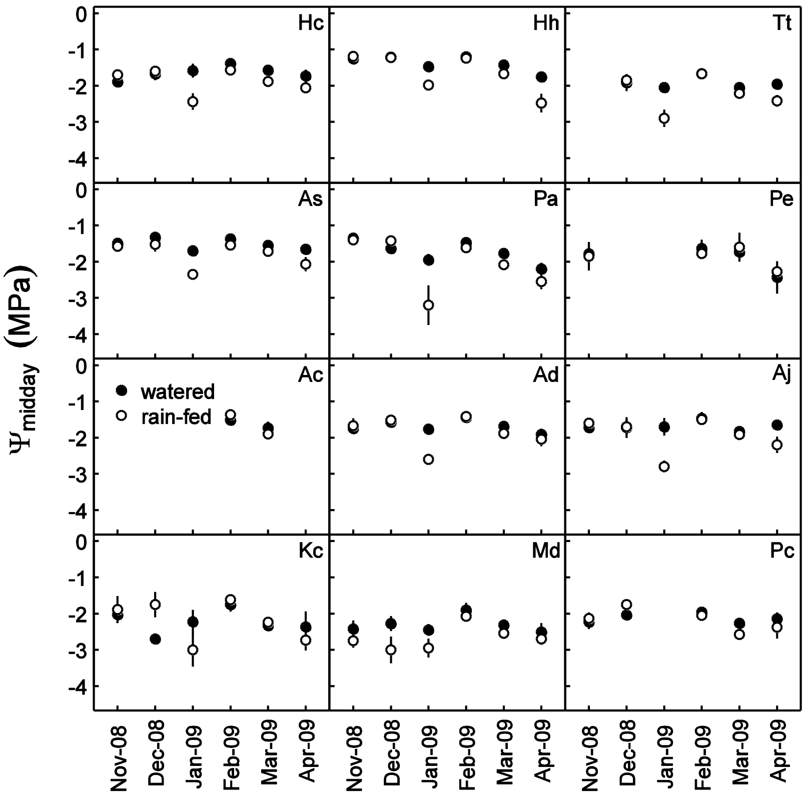

Ψmidday (MPa)
Hc
Hh
Tt
As
Pa
Pe
Ac
Ad
Aj
Kc
Md
Pc
watered
rain-fed
0
-1
-2
-3
-4
Nov-08
Dec-08
Jan-09
Feb-09
Mar-09
Apr-09

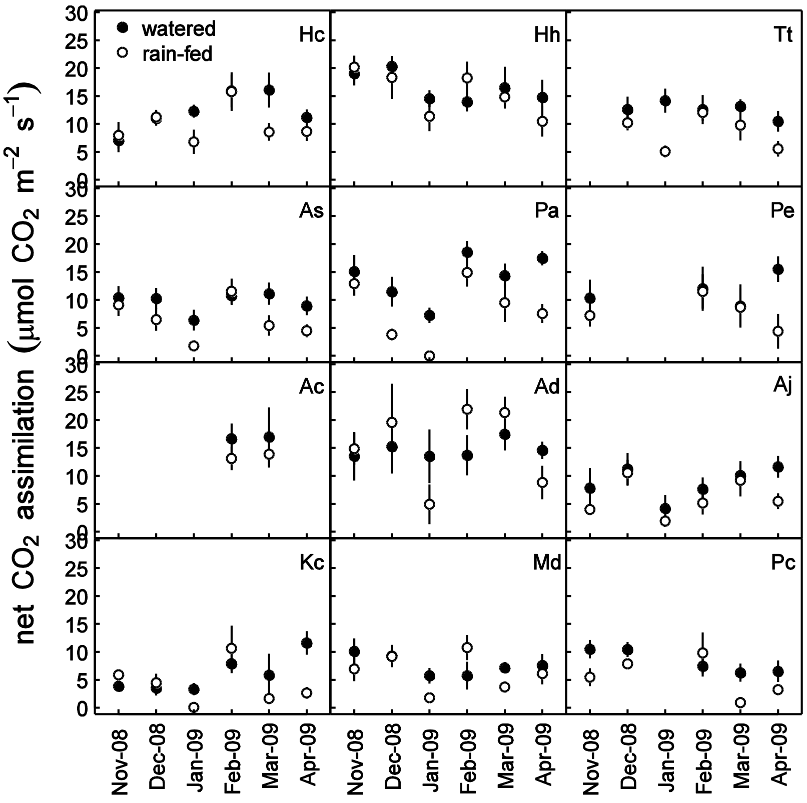

net CO2 assimilation (μmol CO2 m−2 s−1)
watered
rain-fed
Hc
Hh
Tt
As
Pa
Pe
Ac
Ad
Aj
Kc
Md
Pc
0
5
10
15
20
25
30
Nov-08
Dec-08
Jan-09
Feb-09
Mar-09
Apr-09

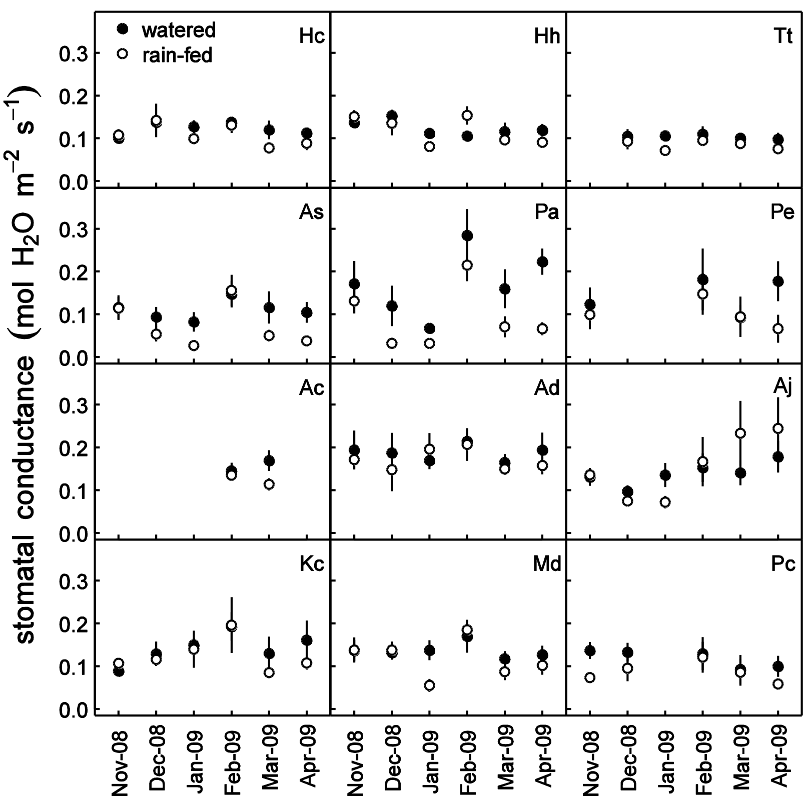

stomatal conductance (mol H2O m−2 s−1)
watered
rain-fed
Hc
Hh
Tt
As
Pa
Pe
Ac
Ad
Aj
Kc
Md
Pc
0.0
0.1
0.2
0.3
Nov-08
Dec-08
Jan-09
Feb-09
Mar-09
Apr-09

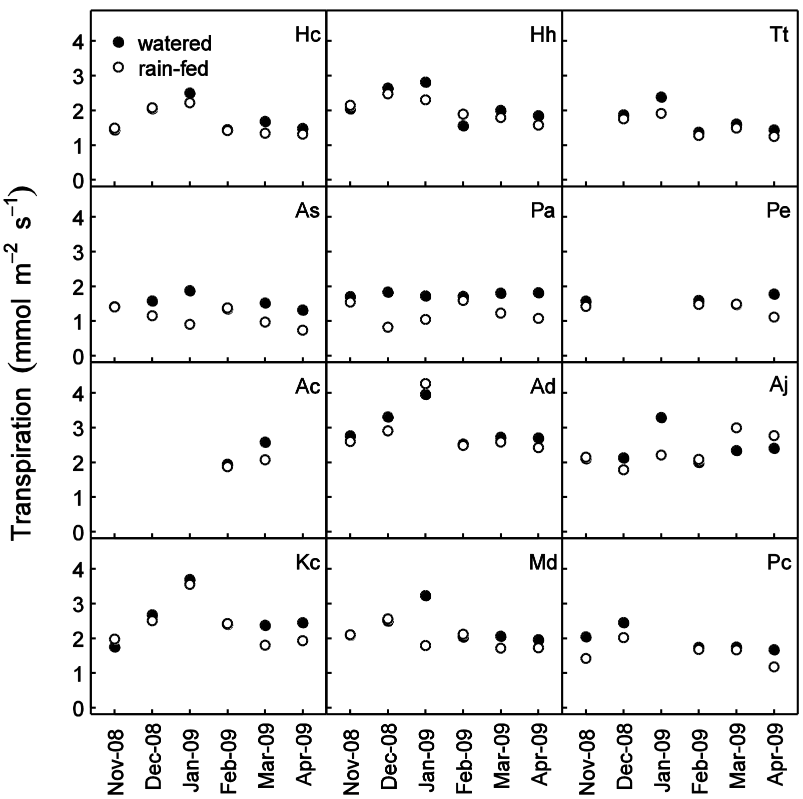
watered
rain-fed
Hc
Hh
Tt
As
Pa
Pe
Ac
Ad
Aj
Kc
Md
Pc
Transpiration (mmol m^{-2} s^{-1})
Nov-08
Dec-08
Jan-09
Feb-09
Mar-09
Apr-09

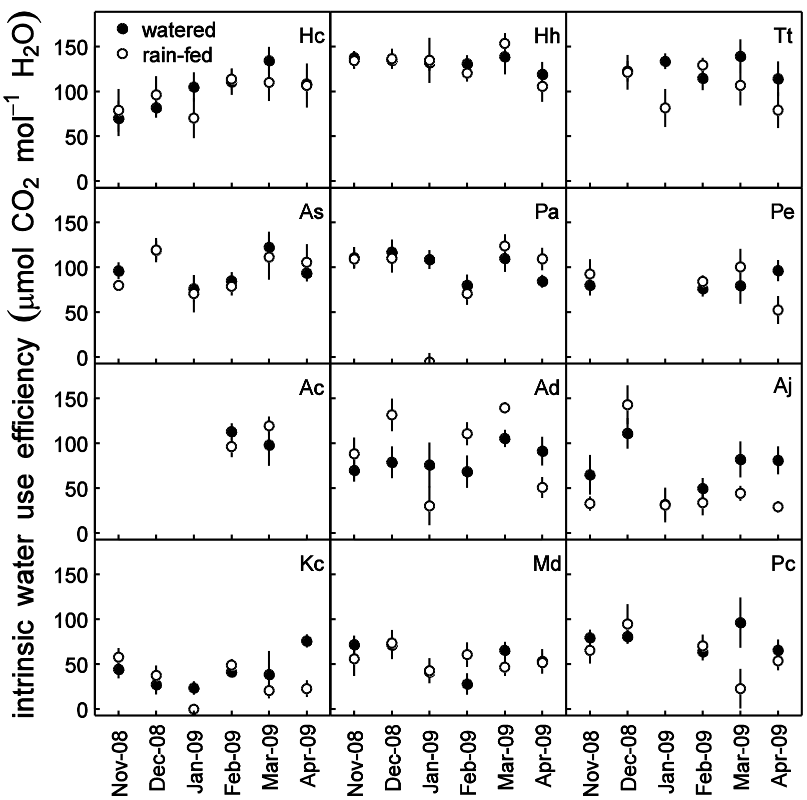
watered
rain-fed
Hc
Hh
Tt
As
Pa
Pe
Ac
Ad
Aj
Kc
Md
Pc
intrinsic water use efficiency (μmol CO_2 mol^{-1} H_2O)
Nov-08
Dec-08
Jan-09
Feb-09
Mar-09
Apr-09

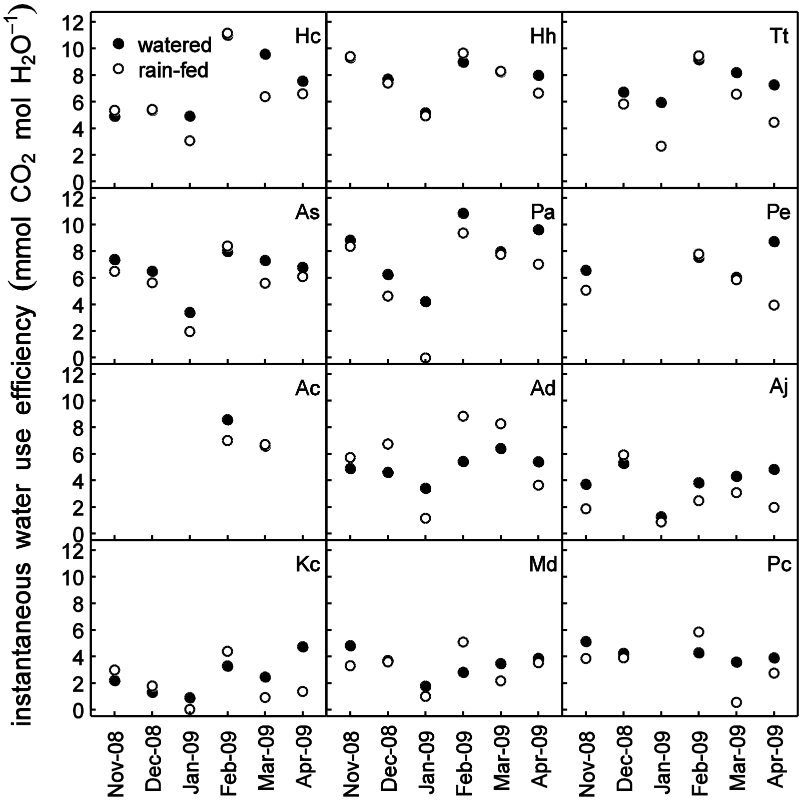
instantaneous water use efficiency (mmol CO_2 mol H_2O^{-1})
watered
rain-fed
Hc
Hh
Tt
As
Pa
Pe
Ac
Ad
Aj
Kc
Md
Pc
0
2
4
6
8
10
12
Nov-08
Dec-08
Jan-09
Feb-09
Mar-09
Apr-09

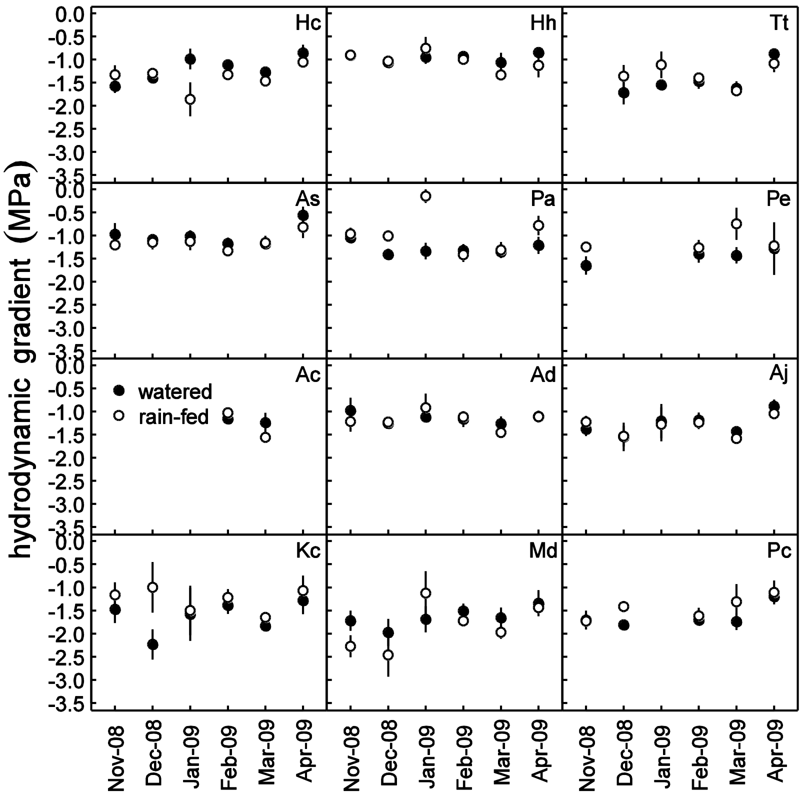
hydrodynamic gradient (MPa)
watered
rain-fed
Hc
Hh
Tt
As
Pa
Pe
Ac
Ad
Aj
Kc
Md
Pc
0.0
-0.5
-1.0
-1.5
-2.0
-2.5
-3.0
-3.5
Nov-08
Dec-08
Jan-09
Feb-09
Mar-09
Apr-09

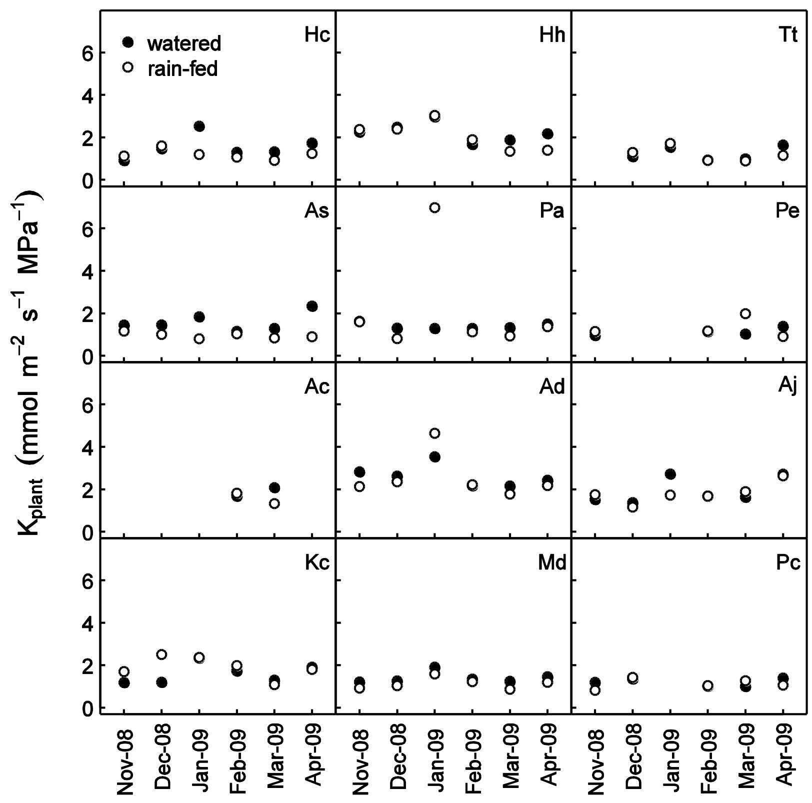
watered
rain-fed
Hc
Hh
Tt
As
Pa
Pe
Ac
Ad
Aj
Kc
Md
Pc
K plant (mmol m-2 s-1 MPa-1)
0
2
4
6
Nov-08
Dec-08
Jan-09
Feb-09
Mar-09
Apr-09
